# Development-associated microRNAs in grains of wheat (*Triticum aestivum* L.)

**DOI:** 10.1186/1471-2229-13-140

**Published:** 2013-09-23

**Authors:** Fanrong Meng, Hao Liu, Ketao Wang, Lulu Liu, Shaohui Wang, Yanhong Zhao, Jun Yin, Yongchun Li

**Affiliations:** 1 College of Life Science, Henan Agricultural University, Zhengzhou 450002, China; 2 National Engineering Research Center for Wheat, Henan Agricultural University, Zhengzhou 450002, China; 3 Collaborative Innovation Center of Henan Grain Crops, Henan Agricultural University, Zhengzhou 450002, China; 4 College of Agriculture, Ludong University, Yantai 264025, China

**Keywords:** MicroRNA, Grain development, Wheat (*Triticum aestivum* L.)

## Abstract

**Background:**

MicroRNAs (miRNAs) are a class of regulatory small RNAs (sRNAs) that down-regulate target genes by mRNA degradation or translational repression. Numerous plant miRNAs have been identified. Evidence is increasing for their crucial roles during plant development. In the globally important crop of wheat (*Triticum aestivum* L.), the process by which grains are formed determines yield and end-use quality. However, little is known about miRNA-mediated developmental regulation of grain production. Here, we applied high-throughput sRNA sequencing and genome-wide mining to identify miRNAs potentially involved in the developmental regulation of wheat grains.

**Results:**

Four sRNA libraries were generated and sequenced from developing grains sampled at 5, 15, 25, and 30 days after pollination (DAP). Through integrative analysis, we identified 605 miRNAs (representing 540 families) and found that 86 are possibly involved in the control of grain-filling. Additionally, 268 novel miRNAs (182 families) were identified, with 18 of them also potentially related to that maturation process. Our target predictions indicated that the 104 grain filling-associated miRNAs might target a set of wheat genes involved in various biological processes, including the metabolism of carbohydrates and proteins, transcription, cellular transport, cell organization and biogenesis, stress responses, signal transduction, and phytohormone signaling. Together, these results demonstrate that the developmental steps by which wheat grains are filled is correlated with miRNA-mediated gene regulatory networks.

**Conclusions:**

We identified 605 conserved and 268 novel miRNAs from wheat grains. Of these, 104 are potentially involved in the regulation of grain-filling. Our dataset provides a useful resource for investigating miRNA-mediated regulatory mechanisms in cereal grains, and our results suggest that miRNAs contribute to this regulation during a crucial phase in determining grain yield and flour quality.

## Background

Endogenous small RNAs (sRNAs) in plants, such as microRNAs (miRNAs) and short-interfering RNAs (siRNAs), were first reported in 2002 [1,2]. Since then, our knowledge about these regulatory molecules has been vastly improved [3]. Plant miRNAs are a class of short (~21-nt) sRNAs produced from non-coding, imperfectly complementary (stem-loop) RNA precursors [4], which can be transcribed by RNA polymerase II [5]. The majority of currently known plant miRNAs was identified via size-selected cloning and sequencing [5-7]. Recently developed high-throughput sequencing strategies have greatly expanded the depth of miRNA cloning coverage [8,9]. In addition to model species, more sRNAs have been identified from crop plants such as rice [10-12], maize [13,14], and wheat [15,16]. Studies of plant miRNAs have indicated that this group of regulatory molecules plays crucial roles in numerous biological processes, e.g., general plant development [17,18] and responses to environmental signals [10,19-21]. Moreover, plant miRNAs and their roles in plant development have been extensively reviewed [3,22].

Seed production is a unique transitional process during the life cycle of higher plants, providing a physical link between parental and progeny sporophytic generations [23]. It is likely that the roles of miRNAs in gene regulation are as critical in maturing seeds as they are in other tissues [24-26]. The millions of short sequence reads that have resulted from next-generation sequencing technologies make that technique explicitly suitable for the profiling of miRNAs. Because of high-throughput sequencing, researchers have been able to identify conserved and novel miRNAs in the seeds of rice [7,11], maize [27], barley [28], and *Brassica napus*[29]. Their presence in those species suggests that miRNA-mediated negative regulation has a crucial role during seed development.

Hexaploid wheat is one of the most valuable cereal crops, occupying 17% of all cultivated land and providing approximately 55% of the carbohydrates consumed by humans worldwide [30]. In addition to being a food source, these grains are used as livestock feed and industrial raw materials, which mainly exploits the endosperm reserves of starch and proteins that account for approximately 80% of a mature seed [31]. Hence, the process by which wheat grains develop directly determines yields and quality of the end product. To improve those traits, researchers must have a keen molecular understanding of the mechanisms that modulate those steps in plant growth. Because little is known about miRNA-mediated regulation in developing wheat grains, our study employed high-throughput sequencing to characterize the miRNAs that potentially participate.

## Results

### Dry matter accumulation and appearance of maturing grain

**Figure 1:**
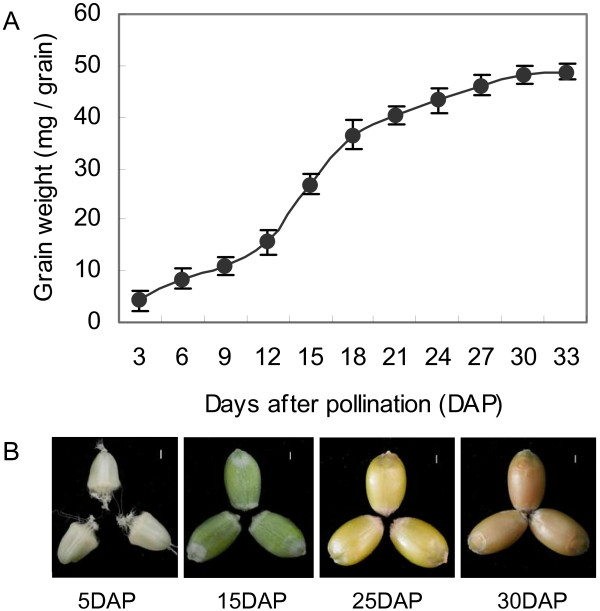
**Weight and appearance of developing wheat grains. A**: patterns of dry matter accumulation; **B**: representative grains at each sampled stage. Scale bars represent 1 mm.

To evaluate the developmental process for wheat grains, we monitored the pattern by which dry matter was accumulated in those tissues. Here, seed weights increased sharply between 12 and 18 days after pollination (DAP) and continued to rise until 30 DAP (Figure 1A). Based on the pattern we observed, we continued our focus mainly on activities between 15 and 25 DAP, the key period for grain-filling, as well as on grains sampled at both 5 DAP and 30 DAP so that we could compare the expression profiles of miRNAs near the beginning and end of that stage of formation. The size and appearance of developing caryopses at those time points are shown in Figure 1B.

### Deep-sequencing of sRNAs in developing grains

**Figure 2:**
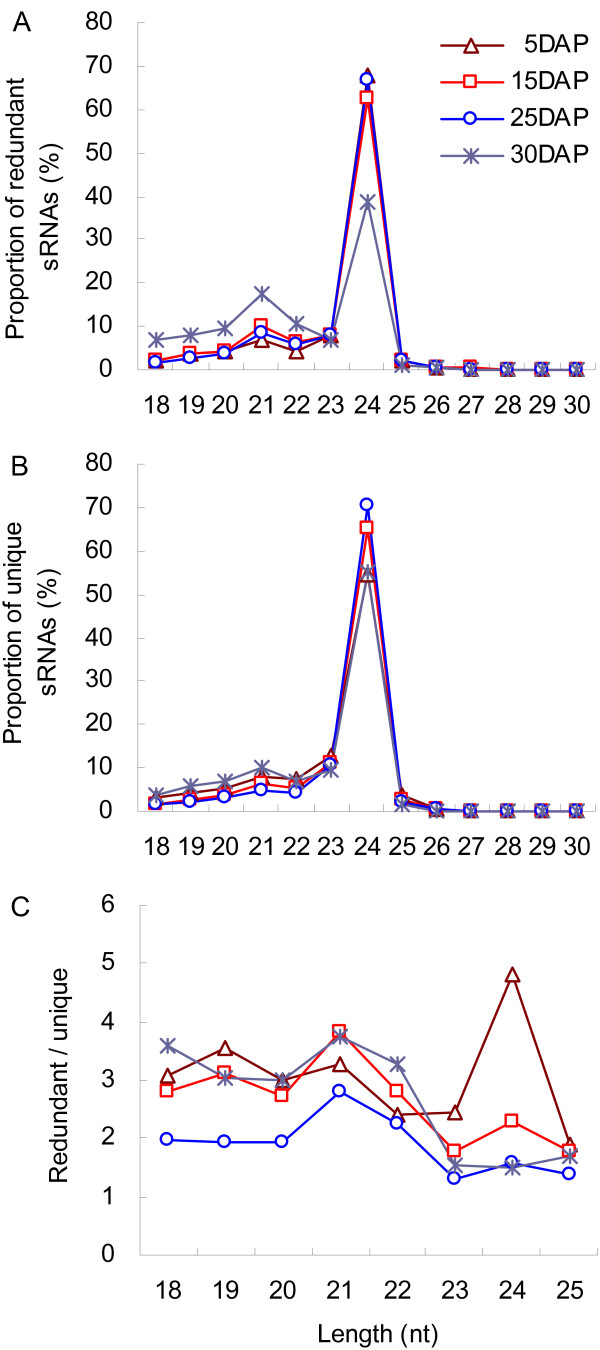
**Distribution of sRNAs detected in developing grains. A**: redundant sRNAs; **B**: unique sRNAs; **C**: ratio of redundant/unique.

To investigate the enrichment of miRNAs, we generated four libraries from developing wheat grains sampled on four dates. After sequencing via the Illumina Genome Analyzer, we removed low-quality reads and corrupted adapter sequences (reads <18 nt or >30 nt long). In all, we obtained 13,525,513 reads (3,492,987 unique) representing 5 DAP, 14,460,398 reads (6,015,357 unique) for 15 DAP, 15,310,251 reads (9,243,757 unique) for 25 DAP, and 13,282,907 reads (6,200,456 unique) for 30 DAP. Our assessment of the size distributions demonstrated that approximately 98% of the detected sRNAs were 18 to 25 nt long, and that 24-nt sRNAs were prevailing in all stages whereas those that were 21 nt long were less abundant (Figure 2A). Further analysis revealed that the distribution of redundant sRNAs in various size classes was quite similar among the libraries from 5, 15, and 25 DAP grains. We found it interesting that the abundance of 24-nt redundant sRNAs was significantly decreased in 30 DAP grains while the proportion of those that were 18 nt to 22 nt were obviously increased (Figure 2A). The distribution patterns for unique sRNAs were similar among all four libraries (Figure 2B). The patterns of distribution between redundant and unique sRNAs indicated that the abundance of sRNAs fluctuated during the period of seed development. Therefore, we evaluated their relative abundance from 18 to 25 nt based on the ratio of redundant/unique and found that expression was generally altered over time (Figure 2C). Transcript levels for most sRNA sizes were obviously decreased in the 25 DAP samples. This was especially true for 24-nt sRNAs, which were gradually repressed. The canonical heterochromatic siRNAs are 24 nt long and these results suggested that siRNA-mediated gene regulation might be involved in the control of grain formation.

**Figure 3:**
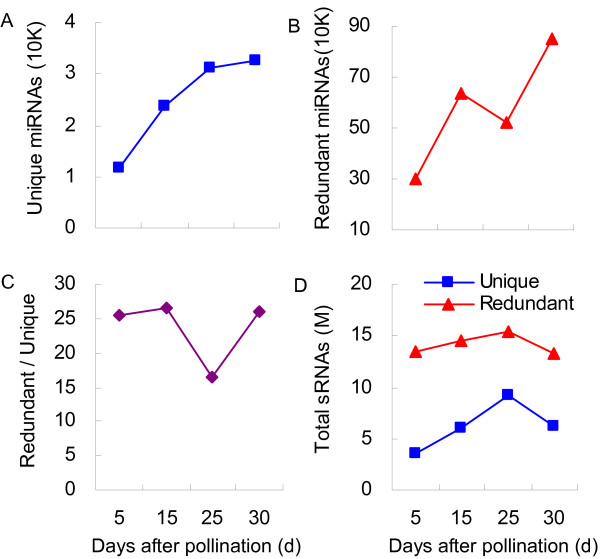
**Enrichment of miRNAs in developing grains. A**: unique miRNAs; **B**: redundant miRNAs; **C**: ratio of redundant/unique; **D**: total sRNAs.

For annotation, we used the sRNA datasets to query the non-coding RNAs deposited in the NCBI GenBank (http://www.ncbi.nlm.nih.gov), Rfam database [32], and miRBase (http://www.mirbase.org/). They were then classified into seven categories: miRNA, rRNA, siRNA, snRNA, snoRNA, tRNA, and those detected but without annotation (Additional file 1). The number of unique miRNAs increased gradually as the process of grain-filling continued (Figure 3A), implying that miRNA-mediated gene silencing is involved in developmental regulation. Moreover, the total read of miRNAs was relatively lower at 25 DAP (Figure 3B), leading to a lower ratio (redundant/unique) of miRNAs at that time point (Figure 3C). Small RNAs were more abundant in the 25 DAP library than in any other (Figure 3D), suggesting that miRNA expression was generally down-regulated at that later stage of maturation.

### Conserved miRNAs differentially expressed in developing grains

**Figure 4:**
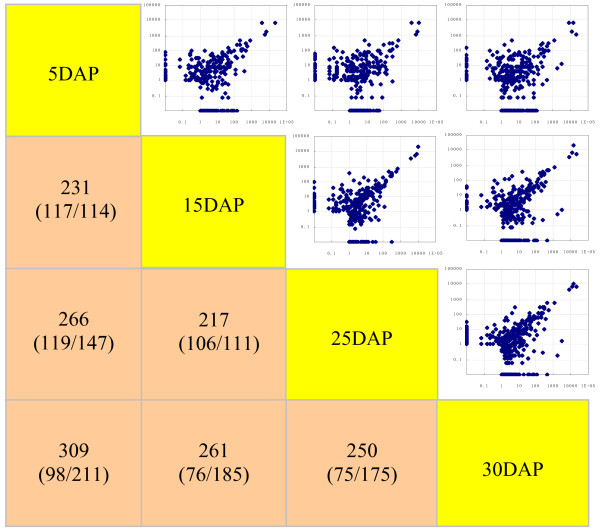
**Scatter plots and comparisons of expression profiles at four developmental stages.** Values represent number of miRNAs differentially repressed/induced between stage-pairings.

To identify the conserved miRNAs in developing grains, we aligned the sRNA sequences with known mature miRNAs from plants in the miRBase. In all, 605 miRNAs representing 540 families were identified (Additional file 2). In general, we found that 424 conserved miRNAs (382 families) were differentially expressed (*p*-values <0.05) over time (Additional file 3). Their scatter plots illustrated the similarities during the stages of grain formation, as well as the broad relationships among miRNA expression profiles from 5 DAP through 30 DAP (Figure 4). However, some differences in profiles were apparent from this examination. When compared with values at 5 DAP, 231, 266, and 309 miRNAs were significantly up- or down-regulated (*p* <0.05) at 15 DAP, 25 DAP, and 30 DAP, respectively. It is likely that the number of up-regulated miRNAs was gradually increased (114, 147, and 211 at 15 DAP, 25 DAP, and 30 DAP, respectively) over time, demonstrating that mechanisms for miRNA-mediated repression are involved in this developmental regulation.

### Conserved miRNAs associated with grain-filling

**Figure 5:**
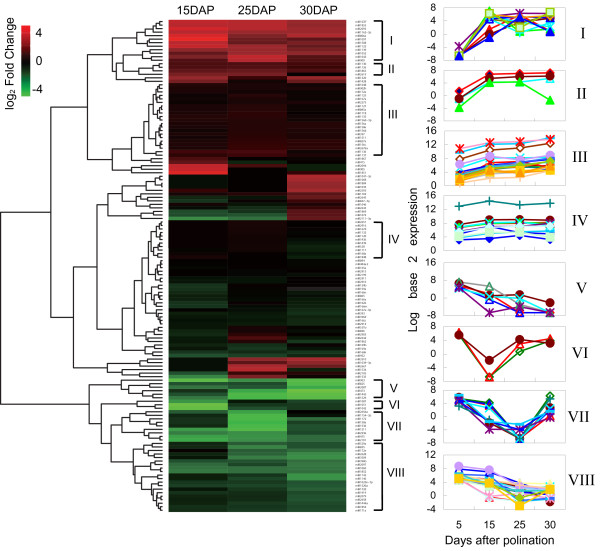
**Hierarchical clustering of 140 conserved miRNAs highly expressed in developing grains.** Clustering was performed based on fold-changes between 5 DAP and 15, 25 or 30 DAP. I-VIII indicate eight clusters of miRNAs which were potentially involved in the regulation of grain filling.

Among those 424 differentially expressed miRNAs, expression was lower for 284 miRNAs, i.e., those with fewer than 20 TPMs (transcripts per million). For our investigation, we selected 140 miRNAs (125 families) highly expressed (at least has a TPM ≥20 in the four stages detected) in developing grains (Additional file 3). To find common expression patterns, we performed hierarchical clustering based on fold-changes in expression from a base line of 5 DAP. In all, 86 miRNAs could be sorted into eight clusters (Figure 5, Additional file 4) that we considered to be potentially involved in the regulation of grain-filling.

The first four clusters (I-IV) of conserved miRNAs generally included those that were up-regulated in the process of grain-filling. Clusters I and II comprised 16 miRNAs with expression that was very low at 5 DAP, but which greatly increased at 15 DAP, and remained high for most miRNAs through 30 DAP. Considering that 15 DAP and 25 DAP are crucial stages during which a complex gene regulatory network is involved in endosperm development, we concluded that those significantly induced miRNA candidates in Clusters I and II might have important regulatory roles. Expression of the miRNAs in Cluster III was gradually increased over time. For example, miR167a, miR156a, and miR156c, were quite abundant, with expression levels at 5 DAP of 1825.73, 845.74, and 212.12 TPM, respectively (Additional file 4). Although they were up-regulated by only a few fold at 15 DAP, they could have a greater regulatory effect because of their more abundant basis. Cluster IV had 11 miRNAs that were only slightly up-regulated. Of these, eight were more highly expressed, with TPM values of >100 at 15 DAP and 25 DAP. For example, the TPMs for miR168a were 7509.36 at 5 DAP and 21776.72 at 15 DAP.

Four clusters of miRNAs showed repression, including six members that were rapidly down-regulated in Cluster V. For example, although miR473 was highly expressed at 5 DAP (TPM = 150.97), its level decreased to 0.13 TPM at 25 DAP and was undetectable in 30 DAP grains (Additional file 4). Clusters VI and VII contained 3 and 9 miRNAs, respectively, all of which were significantly repressed at 15 DAP and/or 25 DAP. Finally, Cluster VIII had 20 members, including miR2628 and miR1146, were gradually repressed over time.

### Novel miRNAs identified in wheat grains

**Figure 6:**
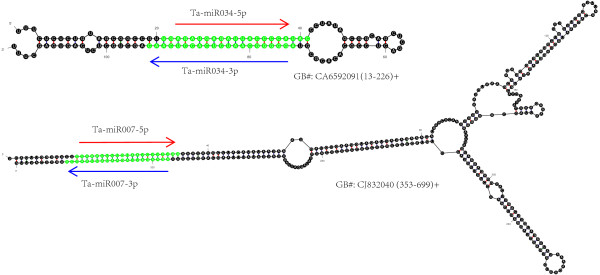
**Predicted stem-loop structures of Ta-MIR034 and Ta-MIR007.** Locations of precursors are indicated in brackets.

Novel miRNAs were predicted according to the characteristic hairpin structures of their precursors, which distinguishes them from other endogenous sRNAs [33,34]. A total of 268 putative novel miRNAs (182 families) were identified (Additional file 5, Additional file 6). Among them, 24 showed at least a relatively higher expression (TPM ≥10) in developing grains (Table 1). We were interested to learn that Ta-miR034-3p and Ta-miR034-5p are homologous to complementary sequences in the same stem-loop (Figure 6). Moreover, Ta-miR007-5p and Ta-miR007-3p were also located in the same stem-loop generated from a wheat EST (GB#: CJ832040).

**Table 1:** Novel miRNAs highly expressed in developing wheat grains

**miRNA**	**Sequence 5′ to 3′**	**Length (nt)**	**Abundance (TPM)**				**Target description (Accession No.)**
			**5DAP**	**15DAP**	**25DAP**	**30DAP**	
**Cluster I**							
Ta-miR023b-5p	UCGCAAAUAAUGGUGGCCCUCG	22	--	6.09	9.21	12.05	Uncharacterized protein (GW667699; TC402735)
Ta-miR128-5p	GUGGAUGAUGAGAUCACAAGUAA	23	--	21.58	29.78	23.56	DRG1 (TC386119); Glycolipid transfer protein (CA648678); Polyol transporter 5 (CA681870).
Ta-miR113-5p	UGGCUACUUCCUUUCCCUUGCC	22	--	22.68	13.52	14.08	bZIP transcription factor (TC448705); Phytosulfokine receptor 1 (TC443080).
Ta-miR021-1-5p	UCUGGCGAGGGACAUACACUGU	22	1.26	61.89	1771.23	7480.74	Protein Rf1 (DR736808); F-box protein PP2-A13-like (TC381152).
**Cluster II**							
Ta-miR018-5p	UCUGUAAACAAAUGUAGGACG	21	2.22	24.55	8.69	59.25	CBL-interacting protein kinase (TC376281); P450 reductase (CK211052); Putative protein kinase (TC404251).
Ta-miR004-1-5p	UCACAAAUAUAAGAUGUUCU	20	2.96	12.72	6.73	16.64	Sucrose-phosphate synthase (TC410332); Nectarin-3 (CA728499); CTD-phosphatase (TC373796); TIF3 (TC398757); receptor-like kinase (TC369729); Lipoyl synthase (TC458824).
Ta-miR034-3p	AGGGGGCAAUCUCACCUCAAC	21	6.21	14.31	8.16	27.18	Serpin-Z2B (BQ243327)
Ta-miR036-3p	UUCCGAAAGGCUUGAAGCAAAU	22	8.87	55.60	21.03	24.99	Light-induced protein (TC387010); SH3 domain protein (DR732608); Aquaporin TIP3-2 (TC390755).
Ta-miR044-1-3p	UGAGAAGGUAGAUCAUAAUAGC	22	3174.59	5490.93	3890.60	9689.90	Mla-like protein (TC368609); NBS-LRR resistance protein (GH723128).
**Cluster III**							
Ta-miR042-3p	UGAUUGAGCCGUGCCAAUAUC	21	--	11.20	--	--	WD and FYVE containing protein 3 (TC418522); Papain-like cysteine proteinase (TC448847).
Ta-miR107-2-3p	AAAAUACUUGUCGGAGAAAUG	21	--	12.66	--	--	Uncharacterized protein (CA664743; DR734972; CK215832)
Ta-miR106-5p	CGGUGGAGCUGGUUGAUGGAC	21	--	141.21	--	--	MYB39 (BE497135); HD-ZIP ROC8 (TC459241); SRG1 (CD454006); alpha-glucosidase (TC393877).
**Cluster IV**							
Ta-miR154-5p	GGCGAGGGACAUACACUGUACA	22	--	--	1772.21	7485.33	Nucleoredoxin (CA605146)
Ta-miR051-3p	AAUAAGUGUGUGAUUGCUACU	21	1.18	--	10.19	15.73	Serine/threonine-protein phosphatase PP2A-4 (TC437472); BONZAI 3 (TC398798).
**Cluster V**							
Ta-miR068-5p	CUCUCUCGGGAGGGCUGAUC	20	14.64	8.58	1.50	1.36	
Ta-miR057-1-3p	UGGCCGUUGGUAGAGUAGGAGA	22	67.80	13.28	2.74	2.63	hypothetical protein (TC415409); ribosomal protein S14 (TC421110).
Ta-miR007-5p	CUUAAUUUUGUAAUCUUCUGG	21	109.64	149.93	--	--	NBS-LRR protein (TC426546); superkiller viralicidic activity 2-like 2 (TC441282).
Ta-miR007-3p	AGAAGAUUAGAAGAUUAAGCA	21	666.30	1234.41	--	--	V-type proton ATPase (TC454891); 2-hydroxy-6-oxo-6-phenylhexa-2,4-dienoate hydrolase (CV759216).
**Cluster VI**							
Ta-miR158-3p	AAGACAACUAAUUUGGGACGG	21	--	--	--	12.72	pre-mRNA-splicing factor (TC435842); receptor protein kinase (TC390384).
Ta-miR159-3p	UGUAGAAAUAGGCACCGGUGC	21	--	--	--	14.83	ATP sulfurylase (TC415167); DCN1 protein (TC382978); acetylglucosaminyltransferase (TC385422).
Ta-miR033-3p	UCAAAGGAUGAGCAAAUACU	20	1.77	3.94	--	10.09	Adenosylhomocysteinase (TC437024); met-10+ protein (TC423089); chaperonin (TC384436).
Ta-miR053-3p	AGGUGGUUAGGAUACUCGGCU	21	1.85	4.15	1.83	10.62	Acetyl-CoA carboxylase (CK153030)
Ta-miR039-5p	CAGAACCAGAAUGAGUAGCUC	21	18.93	--	21.23	--	NAC (TC389150); Elongation factor 1 (TC380217); Ubiquitin-protein ligase (GH729553).
Ta-miR034-5p	UGAGAUGAGAUUACCCCAUAC	21	85.17	75.79	56.76	163.67	F-box domain containing protein (TC411563); hypothetical protein (CJ660567).

**Figure 7:**
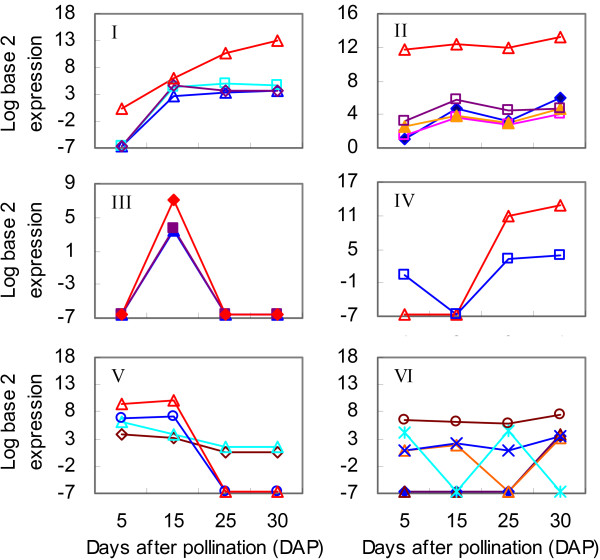
**Expression patterns of novel wheat miRNAs in Clusters I through VI during grain development.** Abundance of expression was normalized to log base-2 values. I-VI, clusters indicated in Table 1.

Further analysis revealed that these 24 highly expressed novel miRNAs could be sorted into Clusters I through VI (Figure 7, Table 1). Cluster I contained four miRNAs for which expression rose sharply at 15 DAP and was maintained at a high level throughout the process of grain-filling. For example, Ta-miR021-1-5p was strongly up-regulated over time, peaking at 30 DAP (TPM = 7480.74). Expression of the five miRNAs within Cluster II was obviously increased at 15, 25, and 30 DAP when compared with levels detected at 5 DAP.

In Cluster III, Ta-miR042-3p, Ta-miR107-2-3p, and Ta-miR106-5p were highly expressed at 15 DAP, but not at any other developmental stages. By contrast, Ta-miR051-3p and especially Ta-miR154-5p, both in Cluster IV, were significantly up-regulated at 25 and 30 DAP. In Cluster V, four novel miRNAs were down-regulated over time. Ta-miR068-5p and Ta-miR057-1-3p were gradually repressed in developing grains whereas Ta-miR007-5p and Ta-miR007-3p were highly expressed at 5 and 15 DAP, but undetectable at 25 and 30 DAP. This contrast in up- and down-regulation among these 18 novel miRNAs from Clusters I through V demonstrated their important regulatory roles.

Finally, the novel miRNAs included in Cluster VI were either expressed only at 30 DAP (near the completion of the filling stage) or else their transcript abundance fluctuated over time. Therefore, their activity did not seem to be associated with this type of developmental regulation. The exception to this was Ta-miR034-5p, also in Cluster VI, which showed strong expression at all sampled time points.

### Validation of miRNAs in developing grains

**Figure 8:**
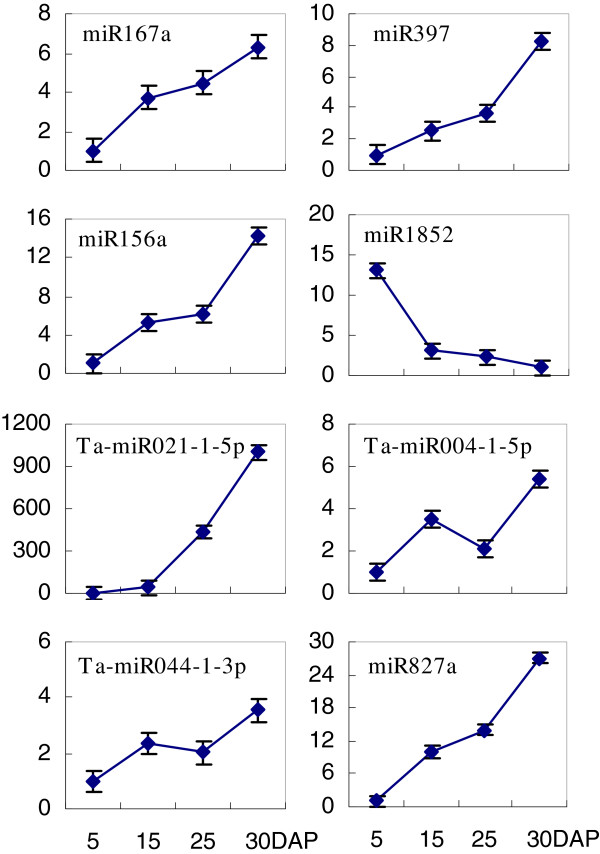
**Verification of expression patterns for 8 miRNAs in developing grains.** DAP, days after pollination. Data for miRNA expression were normalized to endogenous *actin* [GB#: AB181991]. Values are presented as fold-changes (mean ± SD) when compared with levels at 5 DAP. Table 1 Novel miRNAs highly expressed in developing wheat grains.

We conducted quantitative real time PCR (qRT-PCR) to validate the expression patterns of miRNAs identified via high-throughput sequencing. Based on their patterns of development-regulated expression, as determined by deep-sequencing, we selected eight miRNAs for examination. Seven of them -- miR167a, miR397, miR156a, Ta-miR021-1-5p, Ta-miR004-1-5p, Ta-miR044-1-3p, and miR827a -- were predicted to be induced while miR1852 was expected to be repressed. In fact, miR167a, miR397, miR156a, Ta-miR004-1-5p, Ta-miR044-1-3p,and miR827a were significantly up-regulated by 15 DAP and 25 DAP, with expression then peaking at 30 DAP (Figure 8). Their expression profiles were quite similar to those determined by high-throughput sequencing (Figures 5, 7). Although Ta-miR021-1-5p was up- regulated over time while miR1852 was down-regulated, the degree to which their expression was altered was not as dramatic as had been demonstrated with high-throughput sequencing (Additional file 4, Table 1). Those responses may have been affected by the relative abundance of the other sRNAs. Nevertheless, our qRT-PCR results were generally consistent with the data obtained from our high-throughput sequencing, thereby indicating that it is possible to create a set of grain filling-associated miRNAs through deep-sequencing of wheat.

### Targets of grain filling-associated miRNAs

The potential targets of 86 grain filling-associated conserved miRNAs (Additional file 4), as well as 24 highly expressed novel miRNAs (Table 1), were computationally predicted using the *Triticum aestivum* (wheat) unigene library, DFCI Gene Index (TAGI; version 12), and the psRNATarget program (http://plantgrn.noble.org/psRNATarget/) [35]. All of the targets predicted for those conserved and novel miRNAs are shown in Additional file 7 and Additional file 8. Functional annotations of those target genes were performed by BLAST analysis with NCBI and it is found that these grain filling-associated miRNAs potentially target to multiple wheat genes, which including transcription factors, proteins involved in the membrane transporting, ubiquitin-mediated proteolysis, carbohydrate metabolism, responding to stress, signal transduction and phytohormone signaling.

**Figure 9:**
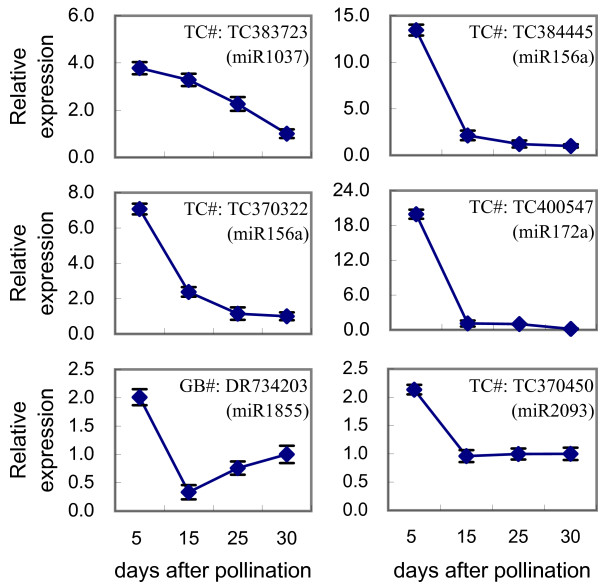
**Expression patterns of selected miRNA targets.** Accession numbers obtained from either DFCI Wheat Gene Index (http://compbio.dfci.harvard.edu/cgi-bin/tgi/gimain.pl?gudb=wheat) (TC#) or GenBank (GB#) are shown. Corresponding miRNAs are indicated in brackets. Data were normalized to endogenous *actin* [GB#: AB181991].

To validate the expression patterns of potential targets for grain filling-associated miRNAs, we selected six target genes (TC383723, TC384445, TC370322, TC400547, DR734203 and TC370450) of candidate miRNAs, which were significantly up-regulated with the development of grains, and gene specific qRT-PCR was performed. The expression analysis demonstrated that all detected target genes were strongly down-regulated during grain-filling (Figure 9), which were negatively correlated to the expression profiles of their corresponding miRNAs (Additional file 4). These results suggested that miRNAs play a crucial regulatory role during the developmental processes of grain-filling in wheat.

## Discussion

For most eukaryotic cells, miRNAs are a class of regulatory sRNAs involved in gene regulation. In plants, some miRNAs play crucial roles in various developmental processes, such as the control of root and shoot architecture, transitions from the vegetative to reproductive phase, and leaf and flower morphogenesis [22,28]. In wheat, 43 miRNAs have been detected in pooled tissue samples (leaves, roots, and spikes) as well as stressed leaves [15,36]. Here, we identified 540 conserved miRNA families and 182 novel miRNA families, with many being differentially expressed during grain-filling in wheat. Despite the growing knowledge about miRNA functions in plants, only those of highly conserved miRNAs, such as miR156 and miR172, have been investigated in crop species. For example, miR156 in rice regulates SPL (Squamosa Promoter-binding protein-Like), which promotes panicle branching and higher grain productivity [37]. Likewise, miR172 regulates AP2-like genes that are involved in controlling floral organ identity in rice, maize, and barley [38-40]. Both of these miRNAs were significantly up-regulated and expressed at high levels in our wheat grains during the filling process. We also predicted several new potential targets for miR156 and miR172, such as MYB-related protein (TC#: TC370322) and the starch negative regulator RSR1 (GB#: CA486144).

The processes by which cereal grains mature strongly influence their yield and flour quality. Formation of the starchy endosperm, which is the greatest contributor to human nutrition, occurs in several stages [40]. In wheat, a major transition point occurs at about 14 d after pollination, essentially marking the end of endosperm cell division and the start of grain-filling (i.e., deposition of starch and gluten proteins) in those cells [31,40]. After approximately 28 d, that deposition of storage reserves decreases and the grains begin to desiccate. To investigate the regulatory mechanisms mediated by miRNAs in wheat, especially those involved in grain-filling, we used high-throughout sequencing for profiling at 5, 15, 25, and 30 DAP, and identified a number of grain filling-associated miRNAs (Additional file 5 and Table 1), which were significantly up- or down- regulated during grain formation. Several grain filling –associated miRNAs identified in this study have also been previously detected in other cereal seeds. For example, miR156a, miR167d, miR168a, and miR172a are significantly up-regulated in barley grains [28]. Additionally, these miRNAs (except miR172a) were also highly expressed in developing seeds of maize [41] and rice [7,11]. Therefore, all of these data suggest that miRNAs play crucial regulatory roles during grain development in cereal crops.

Target prediction indicated that grain filling-associated miRNAs identified in this study potentially target to a set of wheat genes, which are involved in various biological processes including carbohydrate metabolism, protein metabolism, developmental processes, transcription, transport, cell organization and biogenesis, response to stress, signal transduction and phytohormone signaling (Additional file 4 and Table 1). Of those predicted targets, some may be involved in regulating starch accumulation or grain formation. For example, the triose phosphate translocator (TPT), a potential target of miR1855, is an integral membrane protein found in the inner membrane of *Arabidopsis* chloroplasts [42]. There, it is responsible for exporting all of the carbohydrates produced during photosynthesis. Repression of the TPT leads to an increase in starch synthesis in that species. Furthermore, phosphoglycerate kinase (a potential target of miR1037) might participate in regulating carbon-partitioning between starch and sucrose through a series of reactions [43]. In addition, miR172 potentially targets AP2 domain transcription factors CA486144 and CA626451, with the former being a negative regulator of starch production, which is repressed during the middle stages of grain-filling [44]. The expansin gene TC418521, a potential target of miR1137, was highly abundant during early grain expansion but later repressed, implying that levels of expansin are a possible factor in determining grain size [45]. Notably, miR1042 potentially targets a PLAC8 family protein (TC383018), which functions in regulating cell numbers and affects organ size in maize [46]. SNF1-related protein kinase regulatory subunit beta-1 (TC371426), a potential target of miR1211, has vital roles in a signal transduction cascade that controls gene expression and carbohydrate metabolism in higher plants [47]. Its counterpart in barley is thought to modulate the accumulation of storage products during grain-filling [48]. DRG1 (TC386119), a potential target of Ta-miR128-5p, may be crucial to the regulation of either vesicle transport or the activity of enzymes for processing storage proteins [49]. Finally, Ta-miR004-1-5p potentially represses sucrose-phosphate synthase, which is negatively correlated with the accumulation of starch [50]. Overall, our results demonstrate that miRNA-mediated mechanisms operate in the regulation of wheat grain development.

## Conclusions

We identified 104 miRNAs (86 conserved and 18 novel ones) associated with grain-filling from wheat and their potential targets are involved in various biological processes, e.g., carbohydrate and protein metabolism, general plant development, transcription, cellular transport, cell organization and biogenesis, stress responses, signal transduction, and phytohormone signaling. These miRNA-mediated networks play crucial roles during the maturation of wheat grains, and our miRNA data are a valuable resource for future molecular studies that focus on the control of grain development in cereal crops.

## Methods

### Plant materials

Seeds of wheat (*Triticum aestivum* L. cv. Yumai 18) imbibed water for 24 h and were then transferred to a cold chamber (4°C in the dark). After 4 weeks of vernalization, they were planted in pots and cultured in a naturally lit phytotron glasshouse (25°C/20°C day/night). Developing grains were harvested from the middle four rows of a head at 3 to 33 DAP. Each experiment comprised 100 grains, with three replicates. Samples were immediately frozen in liquid nitrogen and stored at −80°C.

### Total RNA isolation

Total RNA was extracted with TRIzol reagent (Invitrogen, Carlsbad, CA, USA) according to the manufacturer’s instructions. Prior to nucleic precipitation, two extra chloroform washes were performed. A 1% agarose gel, stained by ethidium bromide, was run to determine the preliminary integrity of the RNA. All RNA samples were quantified and examined with an ND 1000 spectrophotometer (Nanodrop) for contamination with either protein (A260 nm/A280 nm ratios) or reagent (A260 nm/A230 nm ratios). The RNA integrity number (RIN) was >8 for all samples, as determined with a 2100 Bioanalyzer (Agilent Technologies).

### Construction of sRNA libraries and deep-sequencing

Total RNAs, extracted from developing wheat grains at 5, 15, 25, and 30 DAP, were purified by electrophoretic separations on a 15% TBE-Urea denaturing PAGE gel. The sRNA regions corresponding to 18- to 30-nucleotide bands were excised and recovered. Those sRNAs were then 5′ and 3′ RNA adapter-ligated by T4 RNA ligase. At each step, their lengths were verified before they were purified. The adapter-ligated sRNAs were transcribed into cDNA by Super-Script II Reverse Transcriptase (Invitrogen). We performed PCR-amplifications with primers that annealed to the ends of the adapters. Amplified cDNA constructs were also purified and recovered. The final quality of the cDNA library was ensured by examining its size, purity, and concentration with the 2100 Bioanalyzer. Four libraries, one for each sampling time point, were used for sequencing with the Illumina Genome Analyzer at the Beijing Genomics Institute, Shenzhen, China.

### Analysis of sequencing data

Automated base calling of the raw sequence and vector removal were performed with PHRED and CROSS MATCH programs [51,52]. Any low-quality reads as well as reads with adaptor contamination that were not ligated to any other sequences were discarded. The remaining, high-quality, sequences were trimmed of their adapter sequences; only sequences between 18 nt and 30 nt were analyzed further. The clean reads were mapped to all of the wheat EST sequences obtained from NCBI by SOAP2 (http://soap.genomics.org.cn/). They were aligned to known miRNA precursors (http://www.mirbase.org/) to determine how many identified miRNAs were present. The remaining sequences were used in a BLASTN search of the Rfam database (http://rfam.sanger.ac.uk/) to remove most of the non-siRNA and non-miRNA sequences. Putative origins for the remaining sequences were identified by a BLASTN search against the wheat EST database from NCBI. The protein-coding EST sequences were removed and the remaining non-coding candidate ESTs, with perfect matches to sRNA sequences, were used for fold-back via Mireap (http://sourceforge.net/projects/mireap) based on critical criteria described by Meyers et al. [33]. The secondary structure of precursors were predicted by the Mfold web server (http://mfold.rna.albany.edu/?q=mfold/RNA-Folding-Form) [53].

### Expression analysis of miRNAs based on deep-sequencing data

The frequency of miRNAs was normalized according to the expression of transcripts per million (TPM). For each sample, TPM = (Actual miRNA count/Total count of clean reads) × 10^6^. The fold-change in miRNA expression at 15, 25, and 30 DAP versus 5 DAP was calculated as Fold-change = log base 2 (treatment ⁄ control), where 5 DAP served as the control. All *p*-values were determined according to the following formula:

$$p\left(x\left|y\right)\right)={\left(\frac{N_{2}}{N_{1}}\right)}^{y}\frac{\left(x+y\right)!}{x!y!{\left(1+\frac{N_{2}}{N_{1}}\right)}^{\left(x+y+1\right)}}\begin{array}{c} C\left(y\leq y_{\min}\left|x\right)\right)=\sum_{y=0}^{y\leq y\min}p\left(y\left|x\right)\right)\\ D\left(y\geq y_{\max}\left|x\right)\right)=\sum_{y\geq y\max}^{\infty }p\left(y\left|x\right)\right) \end{array}$$

### Prediction and annotation of potential miRNA targets

Predictions of putative targets were performed by using the most abundant miRNA variants as queries against the TAGI database at psRNATarget (http://plantgrn.noble.org/psRNATarget/) [35]. The following default parameters were applied: maximum expectation value, 3; target accessibility – allowed maximum energy to unpair the target site (UPE), 25; flanking length around the target site for target accessibility analysis, 17 bp upstream and 13 bp downstream; and the range of central mismatch leading to translational inhibition, 9 to 11 nt. To acquire a better annotation of the potential targets, we performed BLAST searches for their sequences against the NCBI database.

### Expressing validation of miRNAs and their targets

We verified the patterns of expression by five conserved wheat miRNAs -- miR167a, miR397, miR156a, miR1852, and miR827a – plus three novel miRNAs -- Ta-miR021-2-5p, Ta-miR004-1-5p, and Ta-miR044-1-3p. A One Step PrimeScript® miRNA cDNA Synthesis Kit (Perfect Real Time; TaKaRa) was used for the RT reactions. The temperature program was adjusted to run for 60 min at 37°C, 5 s at 85°C, and then 4°C. For each miRNA, three biological replicates were performed. qRT-PCR was conducted on a Bio-Rad IQ5 Real-Time PCR Detection System. Each reaction included 2 μL of product from the diluted RT reactions, 1.0 μL of each primer (forward and reverse), 12.5 μL of SYBR® Premix Ex Taq™ (Perfect Real Time; TaKaRa), and 8.5 μL of nuclease-free water. The reactions were incubated in a 96-well plate at 95°C for 30 s, followed by 40 cycles of 95°C for 5 s, 60°C for 30 s, and 72°C for 30 s. All reactions were run in three replicates for each sample. The *actin* gene (GB#: AB181991) served as the endogenous control. We also selected six target genes -- TC383723, TC384445, TC370322, TC400547, TC370450, and DR734203 -- for grain filling-induced miR1037, miR156a, miR172a, miR1855, and miR2093 in order to validate their expression profiles in developing grains via qRT-PCR. All primers are listed in additional file 9.

## Abbreviations

DAP: Days after pollination; miRNAs: microRNAs; qRT-PCR: Quantitative reverse transcription-polymerase chain reaction; siRNAs: Short-interfering RNAs; TPM: Transcripts per million.

## Competing interests

The authors declare that they have no competing interests.

## Authors’ contributions

FM performed the bioinformatics analysis and miRNA validation. HL and KW were involved in preparing the plant materials, deep-sequencing, and characterization of the miRNA targets. LL and SW participated in the expression profiling of miRNA targets. YZ take part in the statistic analylsis. FM, JY, and YL designed and prepared the manuscript. All authors read and approved the final manuscript.

## Supplementary Material

Additional file 1 **Annotation and distribution of sRNAs.** The classifications are listed for all sRNAs detected in the four libraries from developing wheat grains at 5, 15, 25, and 30 DAP. Click here for file

Additional file 2 **Conserved miRNAs identified in developing wheat grains.** Listed sequences and abundances for all conserved miRNAs detected. Click here for file

Additional file 3 **Conserved miRNAs differentially expressed during grain development.** Listed sequences, abundances, as well as ratios of change in expression for differentially expressed miRNAs. Click here for file

Additional file 4 **Conserved miRNAs potentially associated with grain-filling.** Listed sequences, abundances and target descriptions for 86 conserved miRNAs potentially involved in the control of grain-filling. Click here for file

Additional file 5 **Novel miRNAs identified in developing grains.** Listed sequences, abundances and precursors for novel miRNAs identified. Click here for file

Additional file 6 **Secondary structures of 24 highly expressed novel miRNAs.** Secondary structures of 24 highly expressed novel miRNAs. Demonstrated stem-loop structures for novel miRNAs presented in Table 1. Click here for file

Additional file 7 **Predicted targets and annotation for grain filling-regulated conserved miRNAs.** Listed the targets and annotations for conserved miRNAs from Clusters I through VIII, as shown in Figure 5. Click here for file

Additional file 8 **Predicted targets for novel miRNAs.** Listed the targets and annotations for 24 miRNAs highly expressed in developing grains and presented in Table 1. Click here for file

Additional file 9 Primers used in this study. Click here for file

## References

[B1] Llave C Kasschau KD Rector MA Carrington JC Endogenous and silencing-associated small RNAs in plants Plant Cell 2002 13 7 1605 1619 10.1105/tpc.003210 12119378 PMC150710

[B2] Mette MF Van der Winden J Matzke M Matzke AJ Short RNAs can identify new candidate transposable element families in Arabidopsis Plant Physiol 2002 13 1 6 9 10.1104/pp.007047 12226481 PMC1540252

[B3] Chen X Small RNAs in development—insights from plants Curr Opin Genet Dev 2012 13 4 361 367 10.1016/j.gde.2012.04.004 22578318 PMC3419802

[B4] Jones-Rhoades MW Bartel DP Bartel B MicroRNAS and their regulatory roles in plants Annu Rev Plant Biol 2006 13 19 53 10.1146/annurev.arplant.57.032905.105218 16669754

[B5] Xie Z Allen E Fahlgren N Calamar A Givan SA Carrington JC Expression of Arabidopsis MIRNA genes Plant Physiol 2005 13 4 2145 2154 10.1104/pp.105.062943 16040653 PMC1183402

[B6] Reinhart BJ Weinstein EG Rhoades MW Bartel B Bartel DP MicroRNAs in plants Genes Dev 2002 13 13 1616 1626 10.1101/gad.1004402 12101121 PMC186362

[B7] Peng T Lv Q Zhang J Li J Du Y Zhao Q Differential expression of the microRNAs in superior and inferior spikelets in rice (Oryza sativa) J Exp Bot 2012 13 14 4943 4954 10.1093/jxb/err205 21791435

[B8] Lu C Tej SS Luo S Haudenschild CD Meyers BC Green PJ Elucidation of the small RNA component of the transcriptome Science 2005 13 5740 1567 1569 10.1126/science.1114112 16141074

[B9] Chen X Small RNAs - secrets and surprises of the genome Plant J 2010 13 6 941 958 10.1111/j.1365-313X.2009.04089.x 20409269 PMC3062250

[B10] Sunkar R Zhou X Zheng Y Zhang W Zhu JK Identification of novel and candidate miRNAs in rice by high throughput sequencing BMC Plant Biol 2008 13 25 10.1186/1471-2229-8-25 18312648 PMC2292181

[B11] Xue LJ Zhang JJ Xue HW Characterization and expression profiles of miRNAs in rice seeds Nucleic Acids Res 2009 13 3 916 930 10.1093/nar/gkn998 19103661 PMC2647296

[B12] Wang JF Zhou H Chen YQ Luo QJ Qu LH Identification of 20 microRNAs from Oryza sativa Nucleic Acids Res 2004 13 5 1688 1695 10.1093/nar/gkh332 15020705 PMC390330

[B13] Zhai L Liu Z Zou X Jiang Y Qiu F Zheng Y Zhang Z Genome-wide identification and analysis of microRNA responding to long-term waterlogging in crown roots of maize seedlings Physiol Plant 2012 13 2 181 193 22607471 10.1111/j.1399-3054.2012.01653.x

[B14] Ding D Wang Y Han M Fu Z Li W Liu Z Hu Y Tang J MicroRNA transcriptomic analysis of heterosis during maize seed germination PLoS One 2012 13 6 e39578 10.1371/journal.pone.0039578 22761829 PMC3384671

[B15] Yao Y Guo G Ni Z Sunkar R Du J Zhu JK Sun Q Cloning and characterization of microRNAs from wheat (Triticum aestivum L.) Genome Biol 2007 13 6 R96 10.1186/gb-2007-8-6-r96 17543110 PMC2394755

[B16] Xin M Wang Y Yao Y Song N Hu Z Qin D Xie C Peng H Ni Z Sun Q Identification and characterization of wheat long non-protein coding RNAs responsive to powdery mildew infection and heat stress by using microarray analysis and SBS sequencing BMC Plant Biol 2011 13 1 61 10.1186/1471-2229-11-61 21473757 PMC3079642

[B17] Chen X A microRNA as a translational repressor of APETALA2 in Arabidopsis flower development Science 2004 13 5666 2022 2025 10.1126/science.1088060 12893888 PMC5127708

[B18] Zhan S Lukens L Identification of novel miRNAs and miRNA dependent developmental shifts of gene expression in Arabidopsis thaliana PLoS One 2010 13 4 e10157 10.1371/journal.pone.0010157 20405016 PMC2854152

[B19] Fujii H Chiou TJ Lin SI Aung K Zhu JK A miRNA involved in phosphate-starvation response in Arabidopsis Curr Biol 2005 13 22 2038 2043 10.1016/j.cub.2005.10.016 16303564

[B20] Liu PP Montgomery TA Fahlgren N Kasschau KD Nonogaki H Carrington JC Repression of AUXIN RESPONSE FACTOR10 by microRNA160 is critical for seed germination and post-germination stages Plant J 2007 13 1 133 146 10.1111/j.1365-313X.2007.03218.x 17672844

[B21] Sha A Chen Y Ba H Shan Z Zhang X Wu X Qiu D Chen S Zhou X Identification of *Glycine Max* MicroRNAs in response to phosphorus deficiency J Plant Biol 2012 13 4 268 280 10.1007/s12374-011-0255-4

[B22] Chen X Small RNAs and their roles in plant development Annu Rev Cell Dev Biol 2009 13 21 44 10.1146/annurev.cellbio.042308.113417 19575669 PMC5135726

[B23] Meng F Ni Z Wu L Sun Q Differential gene expression between cross-fertilized and self-fertilized kernels during the early stages of seed development in maize Plant Sci 2005 13 1 23 28 10.1016/j.plantsci.2004.07.011

[B24] Martin RC Liu PP Goloviznina NA Nonogaki H MicroRNA, seeds, and Darwin? Diverse function of miRNA in seed biology and plant responses to stress J Exp Bot 2010 13 9 2229 2234 10.1093/jxb/erq063 20335408

[B25] Martin RC Liu P-P Nonogaki H MicroRNAs in seeds: modified detection techniques and potential applications Can J Bot 2006 13 2 189 198 10.1139/b05-141

[B26] Reyes JL Chua NH ABA induction of miR159 controls transcript levels of two MYB factors during Arabidopsis seed germination Plant J 2007 13 4 592 606 10.1111/j.1365-313X.2006.02980.x 17217461

[B27] Li D Wang L Liu X Cui D Chen T Zhang H Jiang C Xu C Li P Li S Deep sequencing of maize small RNAs reveals a diverse set of microRNA in dry and imbibed seeds PLoS One 2013 13 1 e55107 10.1371/journal.pone.0055107 23359822 PMC3554676

[B28] Curaba J Spriggs A Taylor J Li Z Helliwell C miRNA regulation in the early development of barley seed BMC Plant Biol 2012 13 120 10.1186/1471-2229-12-120 22838835 PMC3443071

[B29] Zhao YT Wang M Fu SX Yang WC Qi CK Wang XJ Small RNA profiling in two Brassica napus cultivars identifies microRNAs with oil production- and development-correlated expression and new small RNA classes Plant Physiol 2011 13 2 813 823 22138974 10.1104/pp.111.187666 PMC3271769

[B30] Gill BS Appels R Botha-Oberholster AM Buell CR Bennetzen JL Chalhoub B Chumley F Dvorak J Iwanaga M Keller B A workshop report on wheat genome sequencing: International Genome Research on Wheat Consortium Genetics 2004 13 2 1087 1096 10.1534/genetics.104.034769 15514080 PMC1448818

[B31] Wan Y Poole RL Huttly AK Toscano-Underwood C Feeney K Welham S Gooding MJ Mills C Edwards KJ Shewry PR Transcriptome analysis of grain development in hexaploid wheat BMC Genomics 2008 13 121 10.1186/1471-2164-9-121 18325108 PMC2292175

[B32] Griffiths-Jones S Saini HK Van Dongen S Enright AJ miRBase: tools for microRNA genomics Nucleic Acids Res 2008 13 Database issue D154 D158 17991681 10.1093/nar/gkm952 PMC2238936

[B33] Meyers BC Axtell MJ Bartel B Bartel DP Baulcombe D Bowman JL Cao X Carrington JC Chen X Green PJ Criteria for annotation of plant MicroRNAs Plant Cell 2008 13 12 3186 3190 10.1105/tpc.108.064311 19074682 PMC2630443

[B34] Ambros V Bartel B Bartel DP Burge CB Carrington JC Chen X Dreyfuss G Eddy SR Griffiths-Jones S Marshall M A uniform system for microRNA annotation RNA 2003 13 3 277 279 10.1261/rna.2183803 12592000 PMC1370393

[B35] Dai X Zhao PX psRNATarget: a plant small RNA target analysis server Nucleic Acids Res 2011 13 Web Server issue W155 W159 21622958 10.1093/nar/gkr319 PMC3125753

[B36] Xin M Wang Y Yao Y Xie C Peng H Ni Z Sun Q Diverse set of microRNAs are responsive to powdery mildew infection and heat stress in wheat (Triticum aestivum L.) BMC Plant Biol 2010 13 123 10.1186/1471-2229-10-123 20573268 PMC3095282

[B37] Miura K Ikeda M Matsubara A Song XJ Ito M Asano K Matsuoka M Kitano H Ashikari M OsSPL14 promotes panicle branching and higher grain productivity in rice Nat Genet 2010 13 6 545 549 10.1038/ng.592 20495564

[B38] Lauter N Kampani A Carlson S Goebel M Moose SP microRNA172 down-regulates glossy15 to promote vegetative phase change in maize Proc Natl Acad Sci U S A 2005 13 26 9412 9417 10.1073/pnas.0503927102 15958531 PMC1166634

[B39] Nair SK Wang N Turuspekov Y Pourkheirandish M Sinsuwongwat S Chen G Sameri M Tagiri A Honda I Watanabe Y Cleistogamous flowering in barley arises from the suppression of microRNA-guided HvAP2 mRNA cleavage Proc Natl Acad Sci U S A 2009 13 1 490 495 20018663 10.1073/pnas.0909097107 PMC2806734

[B40] Zhu QH Helliwell CA Regulation of flowering time and floral patterning by miR172 J Exp Bot 2011 13 2 487 495 10.1093/jxb/erq295 20952628

[B41] Kang M Zhao Q Zhu D Yu J Characterization of microRNAs expression during maize seed development BMC Genomics 2012 13 360 10.1186/1471-2164-13-360 22853295 PMC3468377

[B42] Walters RG Ibrahim DG Horton P Kruger NJ A mutant of Arabidopsis lacking the triose-phosphate/phosphate translocator reveals metabolic regulation of starch breakdown in the light Plant Physiol 2004 13 2 891 906 10.1104/pp.104.040469 15173568 PMC514124

[B43] Shah N Bradbeer JW The occurrence of chloroplastic and cytosolic isoenzymes of phosphoglycerate kinase in a range of plant species Planta 1994 13 2 232 237 10.1007/BF00201064 24186426

[B44] Kang GZ Xu W Liu GQ Peng XQ Guo TC Comprehensive analysis of the transcription of starch synthesis genes and the transcription factor RSR1 in wheat (triticum aestivum) endosperm Genome 2013 13 2 115 122 10.1139/gen-2012-0146 23517321

[B45] Lizana XC Riegel R Gomez LD Herrera J Isla A McQueen-Mason SJ Calderini DF Expansins expression is associated with grain size dynamics in wheat (Triticum aestivum L.) J Exp Bot 2010 13 4 1147 1157 10.1093/jxb/erp380 20080826 PMC2826655

[B46] Guo M Rupe MA Dieter JA Zou J Spielbauer D Duncan KE Howard RJ Hou Z Simmons CR Cell number Regulator1 affects plant and organ size in maize: implications for crop yield enhancement and heterosis Plant Cell 2010 13 4 1057 1073 10.1105/tpc.109.073676 20400678 PMC2879740

[B47] Martinez-Barajas E Delatte T Schluepmann H De Jong GJ Somsen GW Nunes C Primavesi LF Coello P Mitchell RA Paul MJ Wheat grain development is characterized by remarkable trehalose 6-phosphate accumulation pregrain filling: tissue distribution and relationship to SNF1-related protein Kinase1 activity Plant Physiol 2011 13 1 373 381 10.1104/pp.111.174524 21402798 PMC3091070

[B48] Sreenivasulu N Radchuk V Strickert M Miersch O Weschke W Wobus U Gene expression patterns reveal tissue-specific signaling networks controlling programmed cell death and ABA- regulated maturation in developing barley seeds Plant J 2006 13 2 310 327 10.1111/j.1365-313X.2006.02789.x 16771774

[B49] Etheridge N Trusov Y Verbelen JP Botella JR Characterization of ATDRG1, a member of a new class of GTP-binding proteins in plants Plant Mol Biol 1999 13 6 1113 1126 10.1023/A:1006137221259 10380799

[B50] Kerr PS Huber SC Israel DW Effect of N-source on soybean leaf sucrose phosphate synthase, starch formation, and whole plant growth Plant Physiol 1984 13 2 483 488 10.1104/pp.75.2.483 16663648 PMC1066934

[B51] Sunkar R Zhu JK Novel and stress-regulated microRNAs and other small RNAs from Arabidopsis Plant Cell 2004 13 8 2001 2019 10.1105/tpc.104.022830 15258262 PMC519194

[B52] Sunkar R Girke T Jain PK Zhu JK Cloning and characterization of microRNAs from rice Plant Cell 2005 13 5 1397 1411 10.1105/tpc.105.031682 15805478 PMC1091763

[B53] Zuker M Mfold web server for nucleic acid folding and hybridization prediction Nucleic Acids Res 2003 13 13 3406 3415 10.1093/nar/gkg595 12824337 PMC169194

