# Supplementary material for: Development-associated microRNAs in grains of wheat (Triticum aestivum L.)
Source: BMC Plant Biol. 2013 Sep 23;13:140. doi: 10.1186/1471-2229-13-140 (PMC4015866; doi:10.1186/1471-2229-13-140)
Supplement: Additional file 1 — Annotation and distribution of sRNAs. The classifications are listed for all sRNAs detected in the four libraries from developing wheat grains at 5, 15, 25, and 30 DAP. [file 1471-2229-13-140-S1.doc]

Additional file 1. Annotation and distribution of small RNAs in developing wheat grains

| Developing Stage | Category | Unique sRNAs | | Redundant sRNAs | | Redundant/Unique |
| --- | --- | --- | --- | --- | --- | --- |
| 5DAP | Total | 3492987 | (100%) | 13525513 | (100%) | 3.87 |
|  | miRNA | 11837 | (0.34%) | 300652 | (2.22%) | 25.40 |
|  | rRNA | 47355 | (1.36%) | 543075 | (4.02%) | 11.47 |
|  | siRNA | 256249 | (7.34%) | 2884542 | (21.33%) | 11.26 |
|  | snRNA | 1391 | (0.04%) | 2572 | (0.02%) | 1.85 |
|  | snoRNA | 607 | (0.02%) | 1171 | (0.01%) | 1.93 |
|  | tRNA | 5977 | (0.17%) | 114210 | (0.84%) | 19.11 |
|  | No annotation | 3169571 | (90.74%) | 9679291 | (71.56%) | 3.05 |
| 15DAP | Total | 6015357 | (100%) | 14460398 | (100%) | 2.40 |
|  | miRNA | 23833 | (0.40%) | 632180 | (4.37%) | 26.53 |
|  | rRNA | 59701 | (0.99%) | 875443 | (6.05%) | 14.66 |
|  | siRNA | 166870 | (2.77%) | 1169123 | (8.08%) | 7.01 |
|  | snRNA | 2401 | (0.04%) | 5710 | (0.04%) | 2.38 |
|  | snoRNA | 874 | (0.01%) | 1750 | (0.01%) | 2.00 |
|  | tRNA | 10285 | (0.17%) | 331722 | (2.29%) | 32.25 |
|  | No annotation | 5751393 | (95.61%) | 11444470 | (79.14%) | 1.99 |
| 25DAP | Total | 9243757 | (100%) | 15310251 | (100%) | 1.66 |
|  | miRNA | 31322 | (0.34%) | 519531 | (3.39%) | 16.59 |
|  | rRNA | 46369 | (0.50%) | 503559 | (3.29%) | 10.86 |
|  | siRNA | 180754 | (1.96%) | 616445 | (4.03%) | 3.41 |
|  | snRNA | 3028 | (0.03%) | 8003 | (0.05%) | 2.64 |
|  | snoRNA | 832 | (0.01%) | 1760 | (0.01%) | 2.12 |
|  | tRNA | 9122 | (0.10%) | 262365 | (1.71%) | 28.76 |
|  | No annotation | 8972330 | (97.06%) | 13398588 | (87.51%) | 1.49 |
| 30DAP | Total | 6200456 | (100%) | 13282907 | (100%) | 2.14 |
|  | miRNA | 32602 | (0.53%) | 847560 | (6.38%) | 26.00 |
|  | rRNA | 68503 | (1.10%) | 1719897 | (12.95%) | 25.11 |
|  | siRNA | 80263 | (1.29%) | 479578 | (3.61%) | 5.98 |
|  | snRNA | 3586 | (0.06%) | 10647 | (0.08%) | 2.97 |
|  | snoRNA | 1017 | (0.02%) | 2863 | (0.02%) | 2.82 |
|  | tRNA | 11856 | (0.19%) | 597337 | (4.50%) | 50.38 |
|  | No annotation | 6002629 | (96.81%) | 9625025 | (72.46%) | 1.60 |

DAP, days after pollination of developing wheat grains; Numbers in parentheses indicate the percentage of the unique or redundant small RNAs.
