# Supplementary material for: Development-associated microRNAs in grains of wheat (Triticum aestivum L.)
Source: BMC Plant Biol. 2013 Sep 23;13:140. doi: 10.1186/1471-2229-13-140 (PMC4015866; doi:10.1186/1471-2229-13-140)
Supplement: Additional file 6 — Secondary structures of 24 highly expressed novel miRNAs. Secondary structures of 24 highly expressed novel miRNAs. Demonstrated stem-loop structures for novel miRNAs presented in Table 1. [file 1471-2229-13-140-S6.pdf]

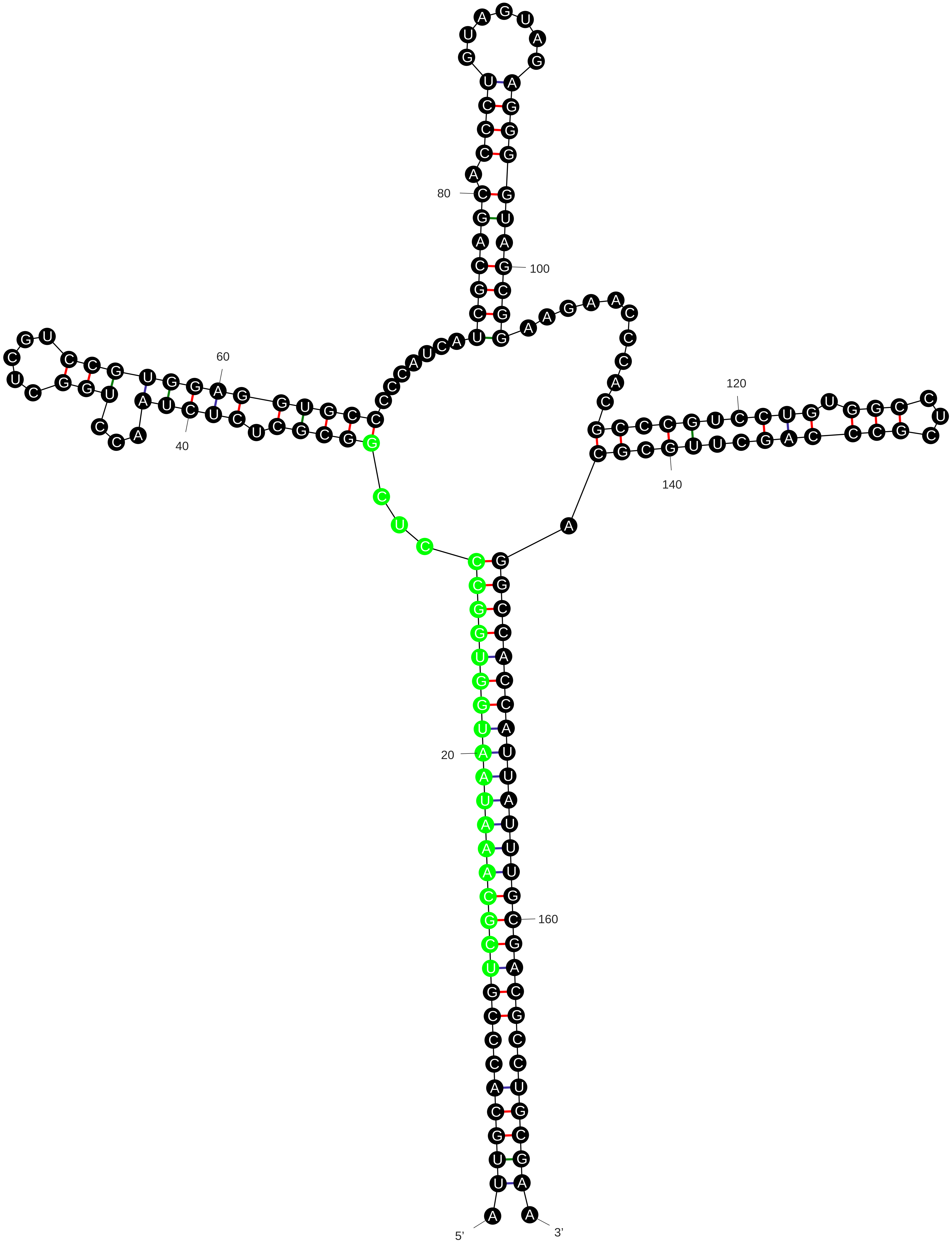

dG = -76.72 [Initially -80.50] Ta-miR023b-5p

Created Sun Apr 21 22:40:53 2013

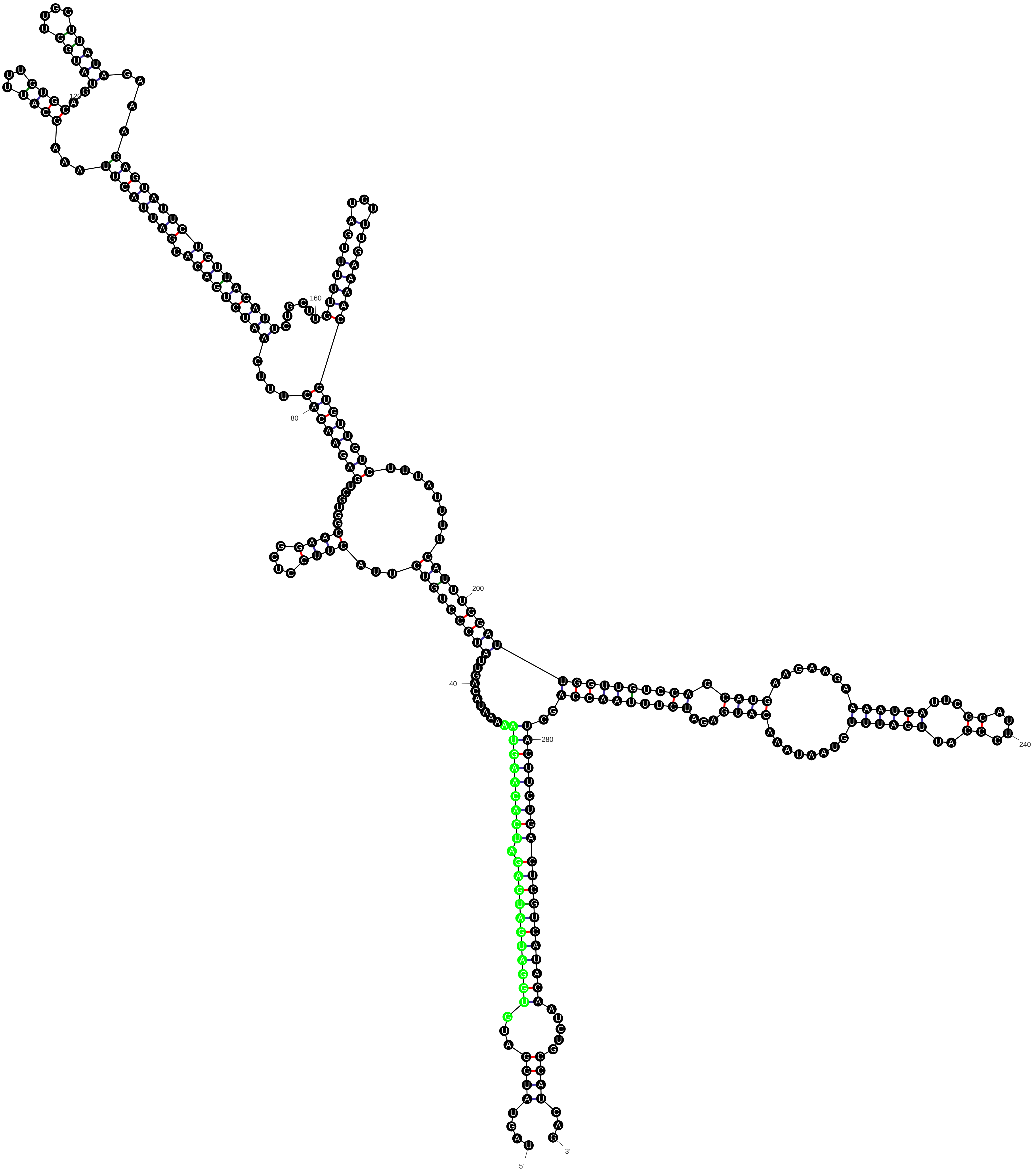

dG = -60.79 [Initially -73.10] Ta-miR128-5p

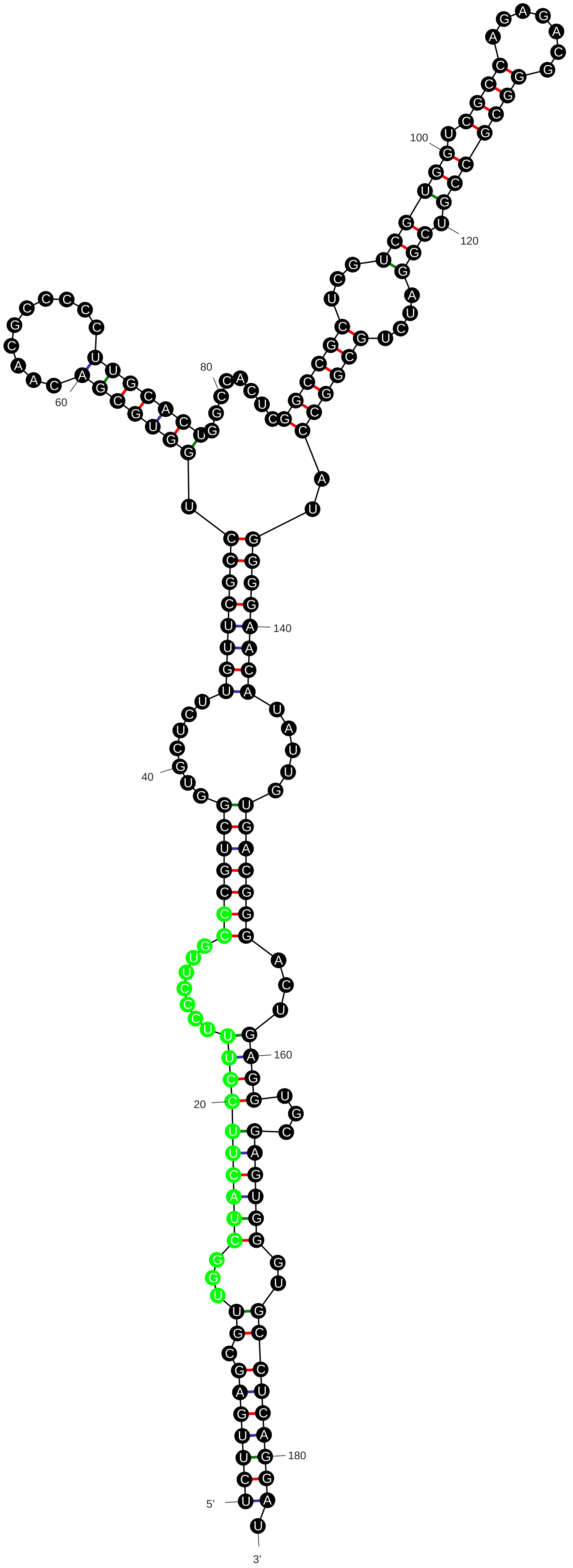

dG = -71.33 [Initially -73.70] Ta-miR113-5p

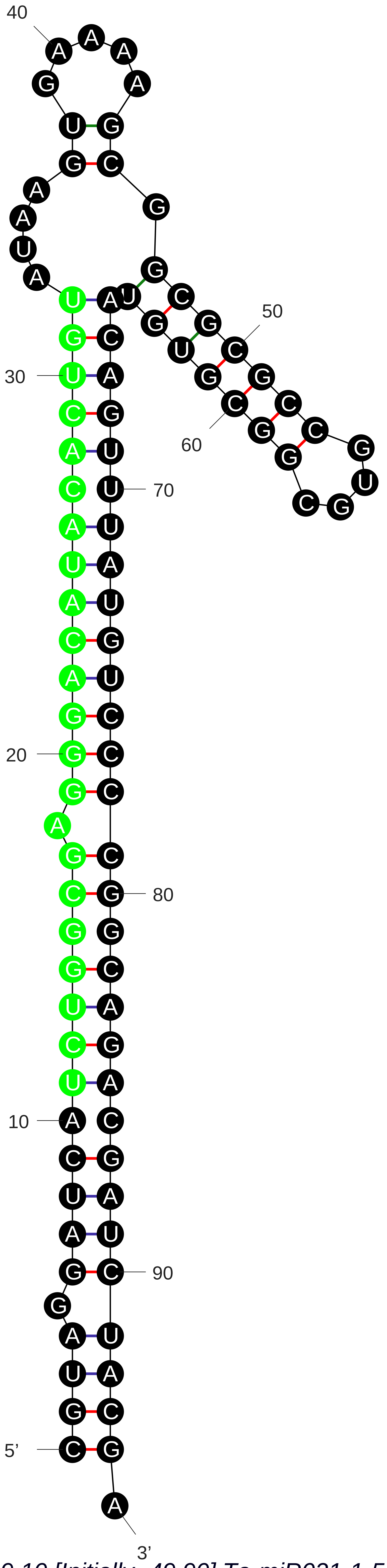

dG = -49.10 [Initially -49.90] Ta-miR021-1-5p

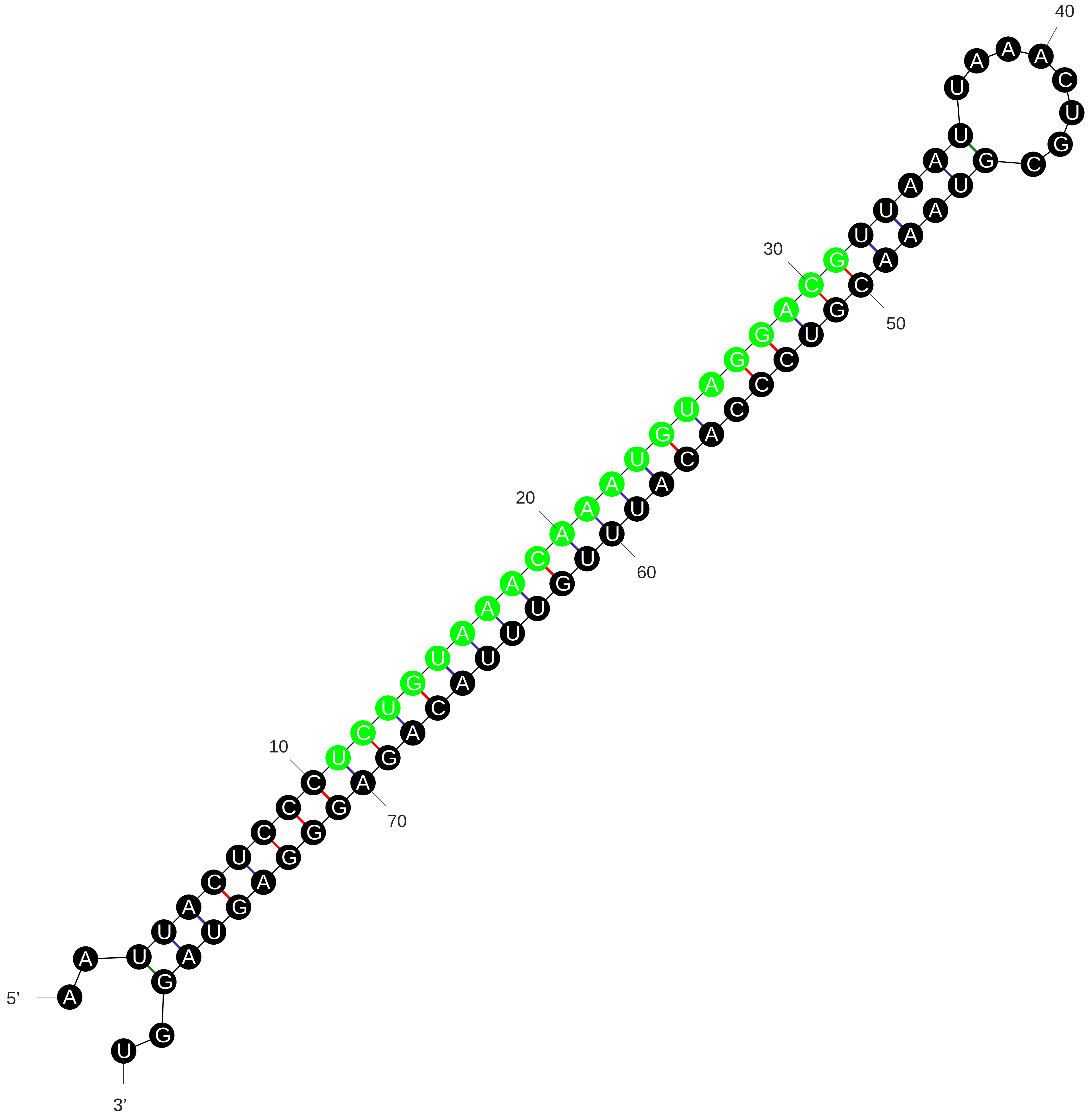

dG = -47.80 [Initially -47.80] Ta-miR018-5p

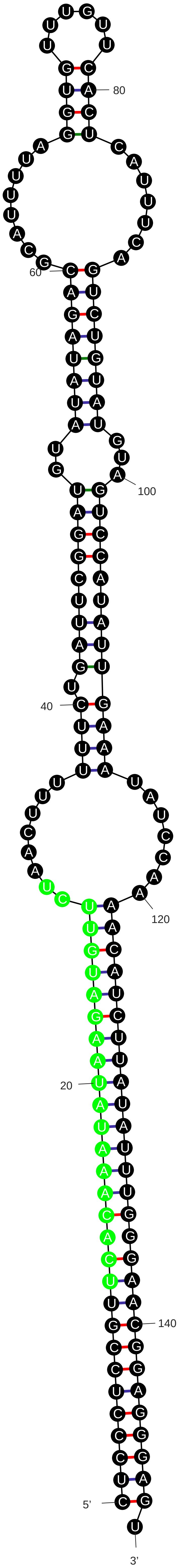

dG = -61.30 [Initially -61.30] Ta-miR004-1-5p

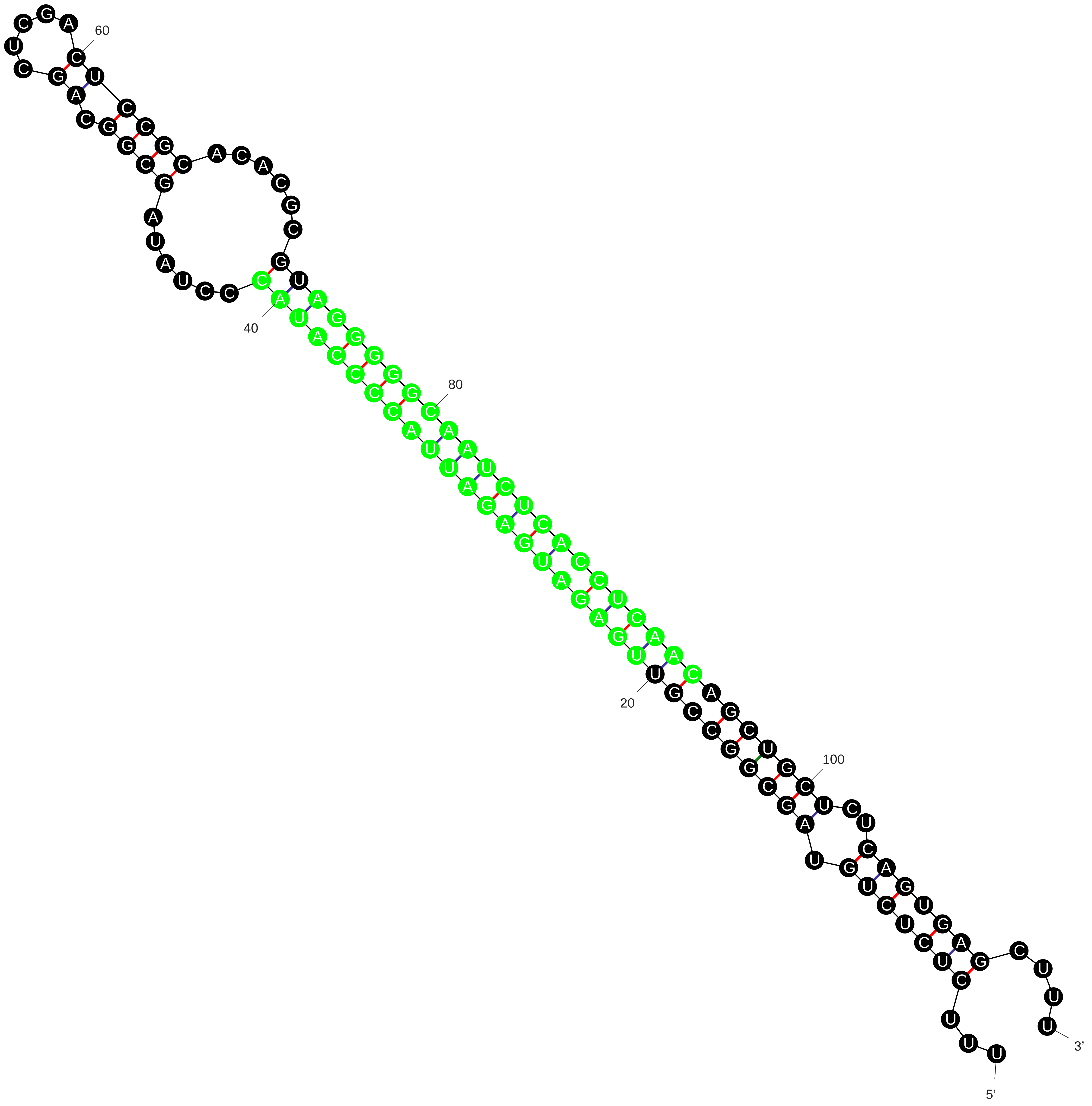

dG = -52.80 [Initially -52.80] Ta-miR034-5p/-3p

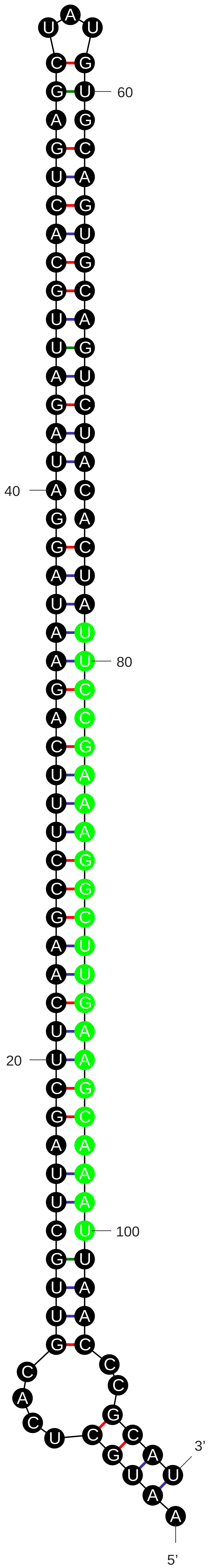

dG = -57.50 [Initially -57.50] Ta-miR036-3p

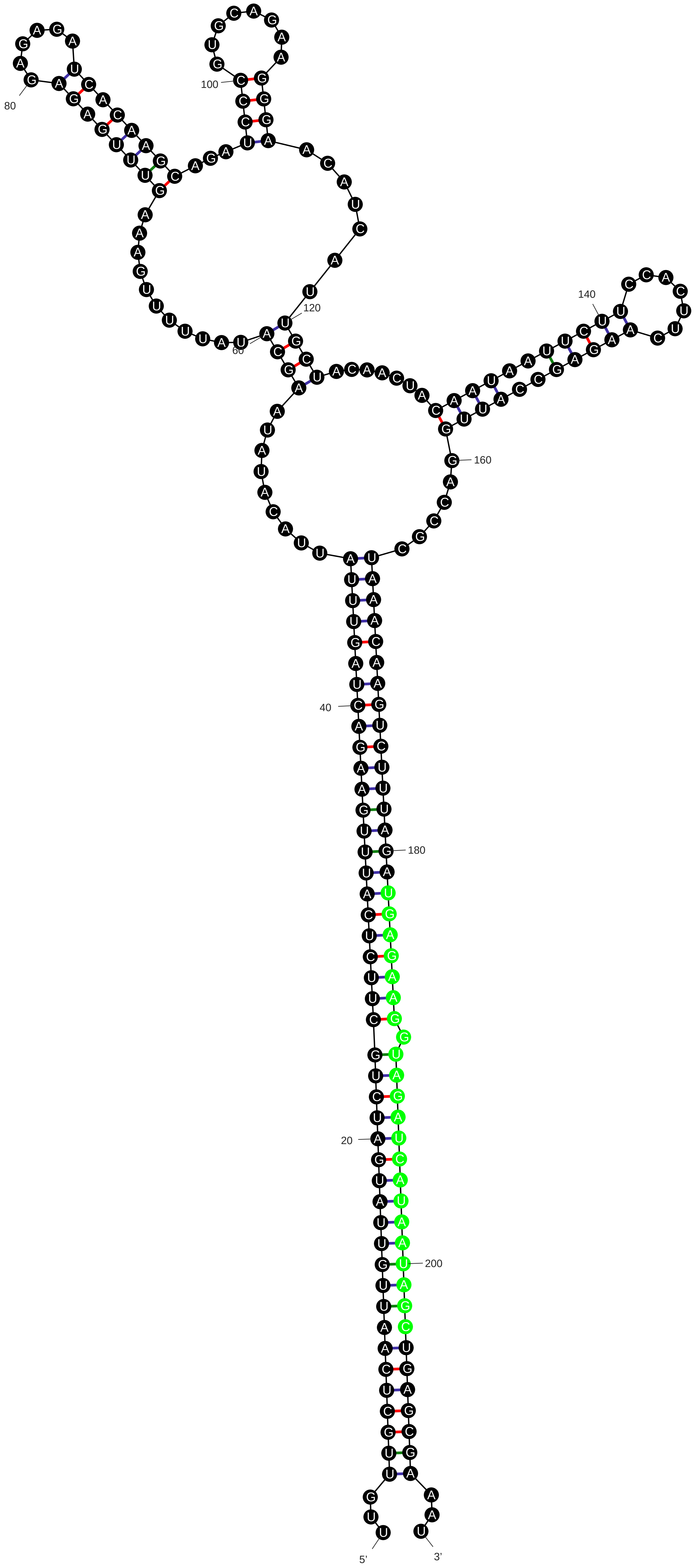

dG = -65.69 [Initially -73.70] Ta-miR044-1-3p

Output of sir_graph (©)
mfold_util 4.6

Created Mon Apr 22 10:02:07 2013

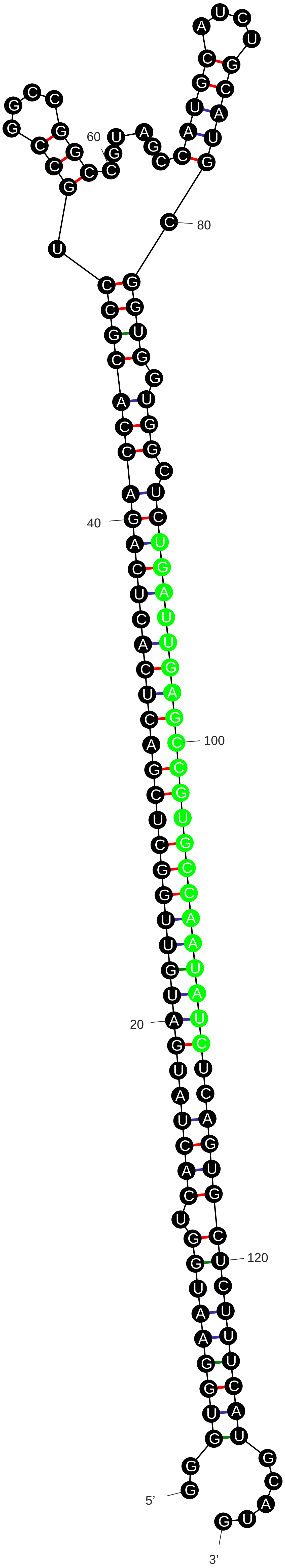

dG = -57.98 [Initially -60.30] Ta-miR042-3p

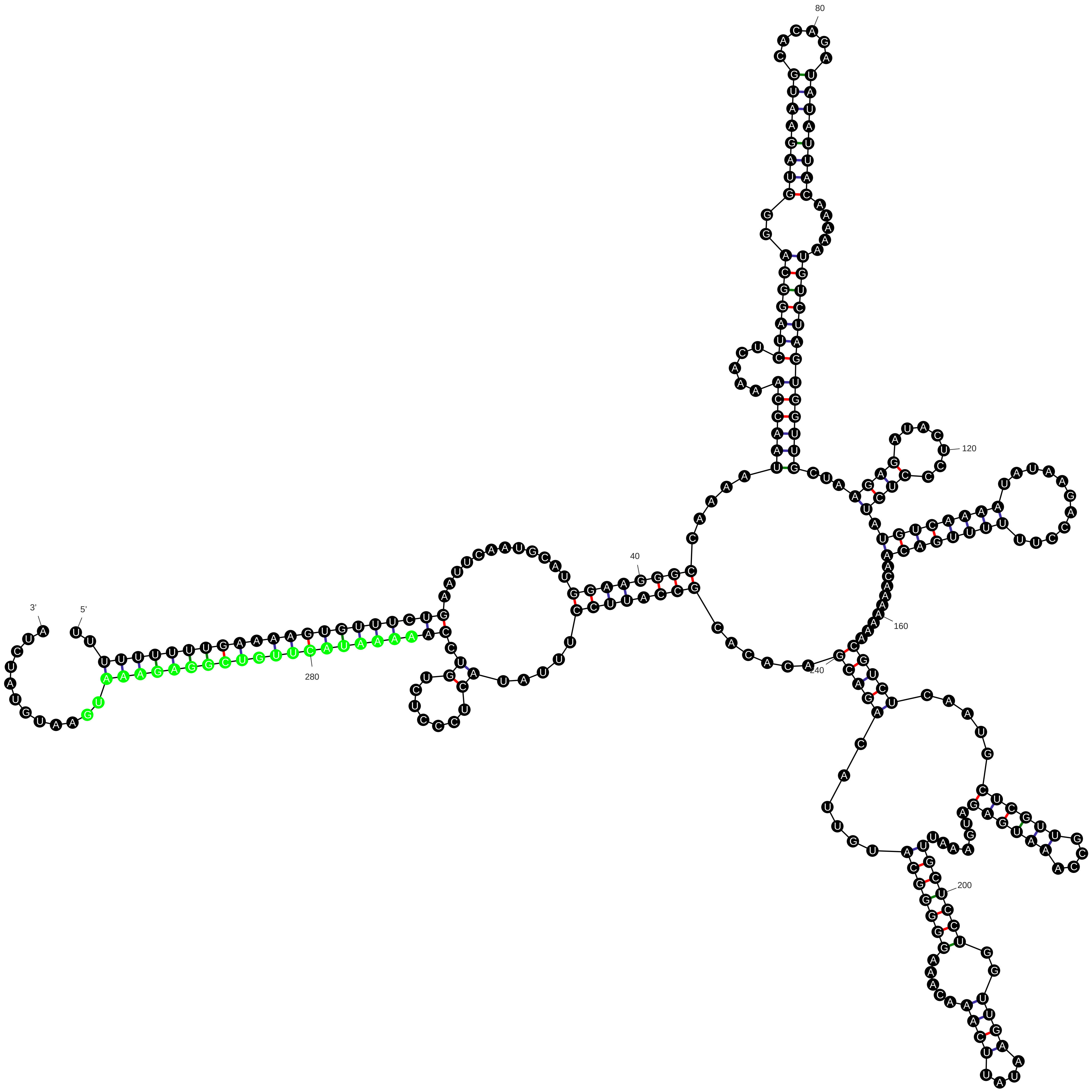

dG = -57.86 [Initially -67.80] Ta-miR107-2-3p

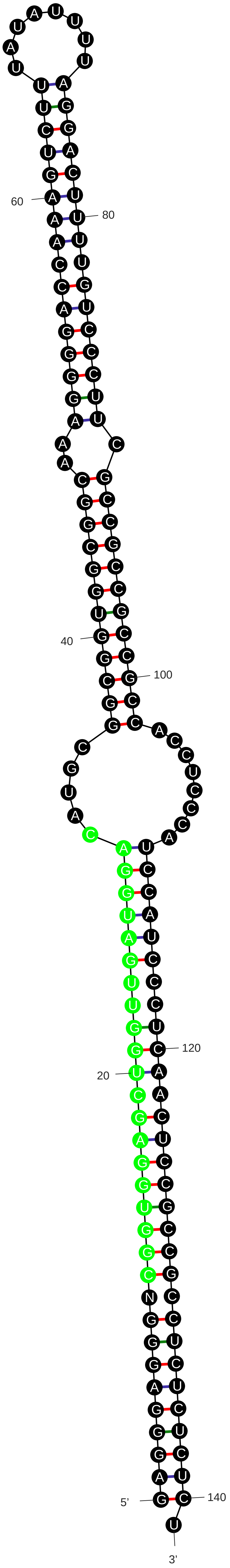

dG = -90.00 [Initially -89.20] Ta-miR106-5p

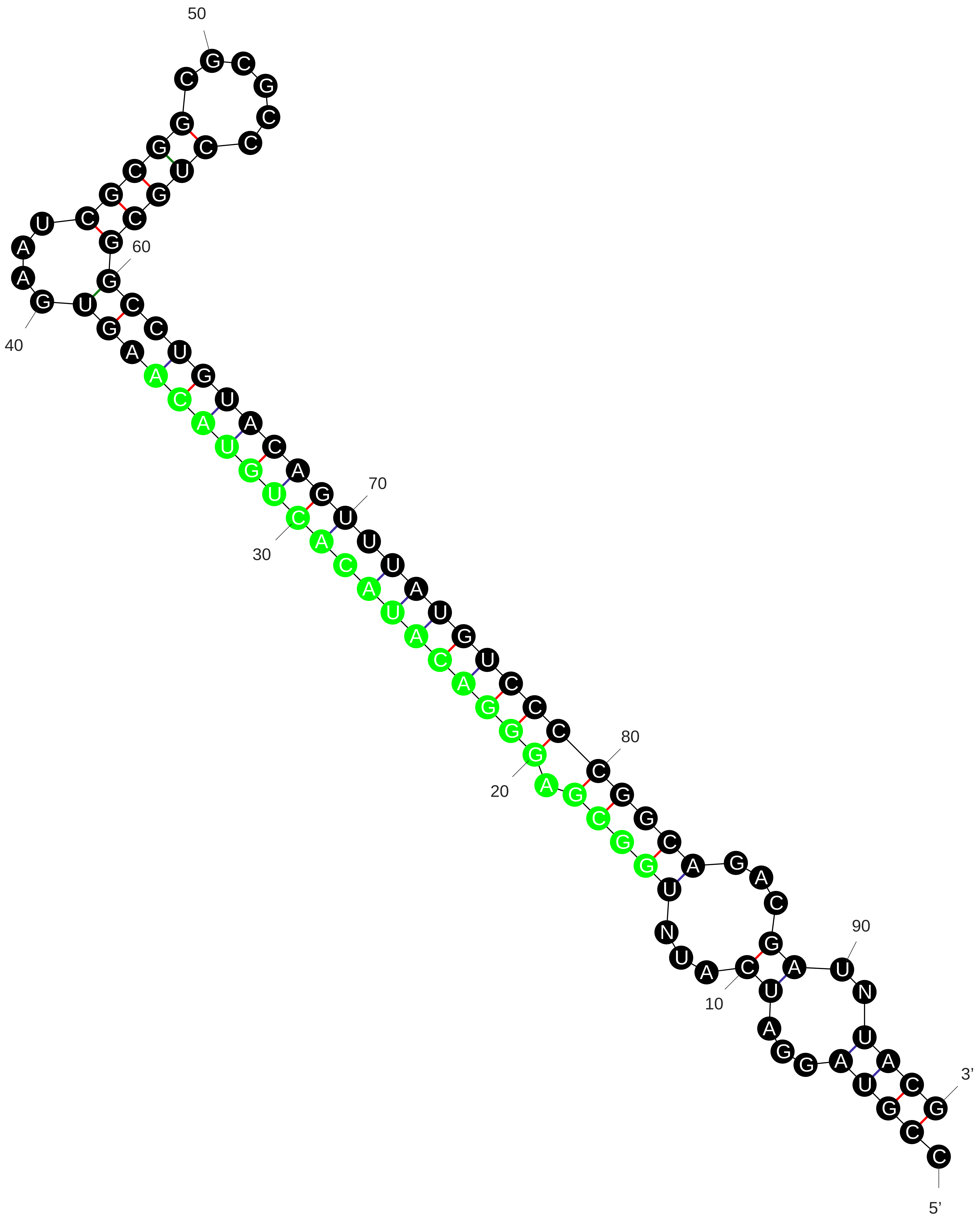

dG = -39.80 [Initially -39.80] Ta-miR154-5p

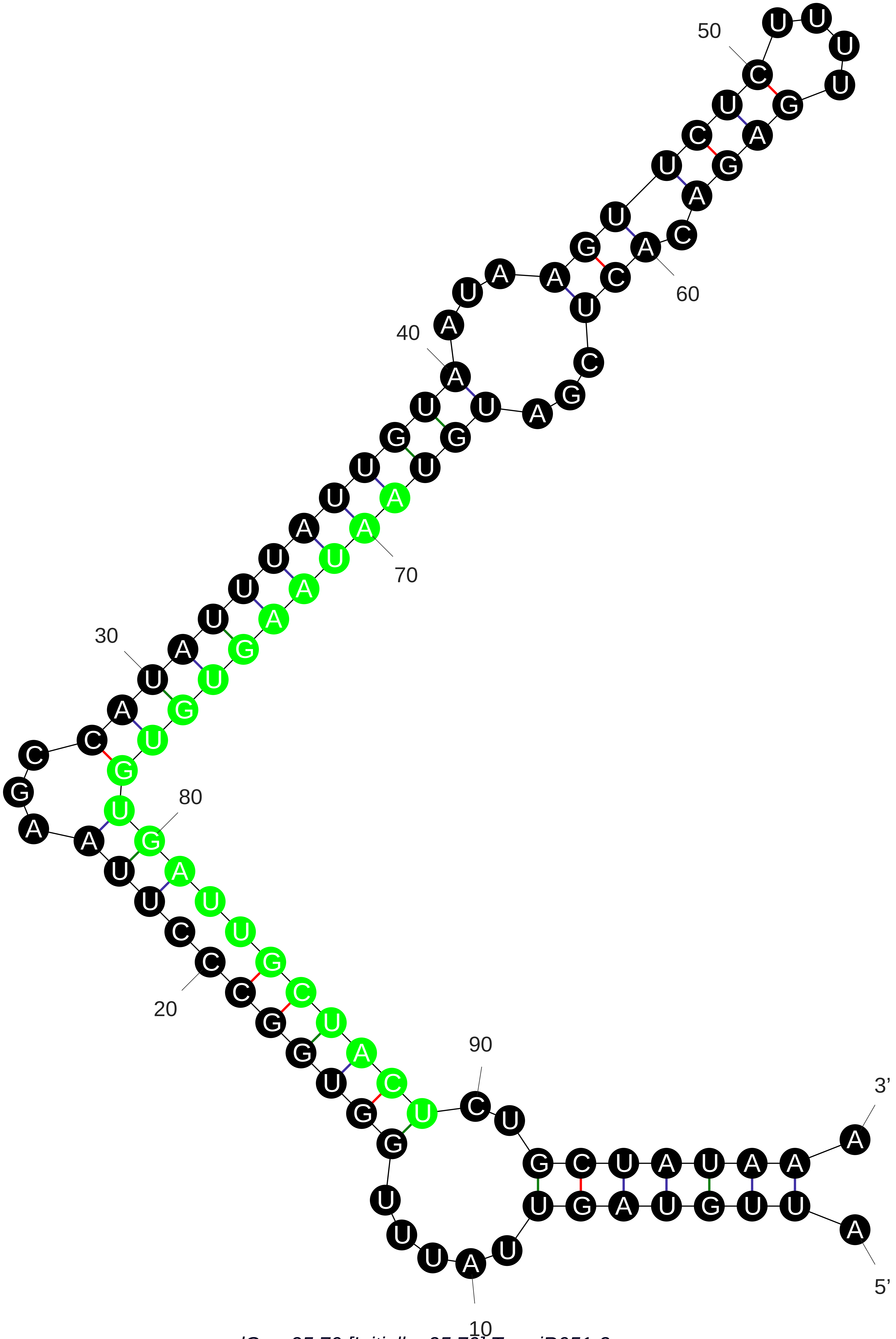

dG = -25.70 [Initially -25.70] Ta-miR051-3p

Output of sir_graph (©)
mfold_util 4.6

Created Mon Apr 22 10:11:38 2013

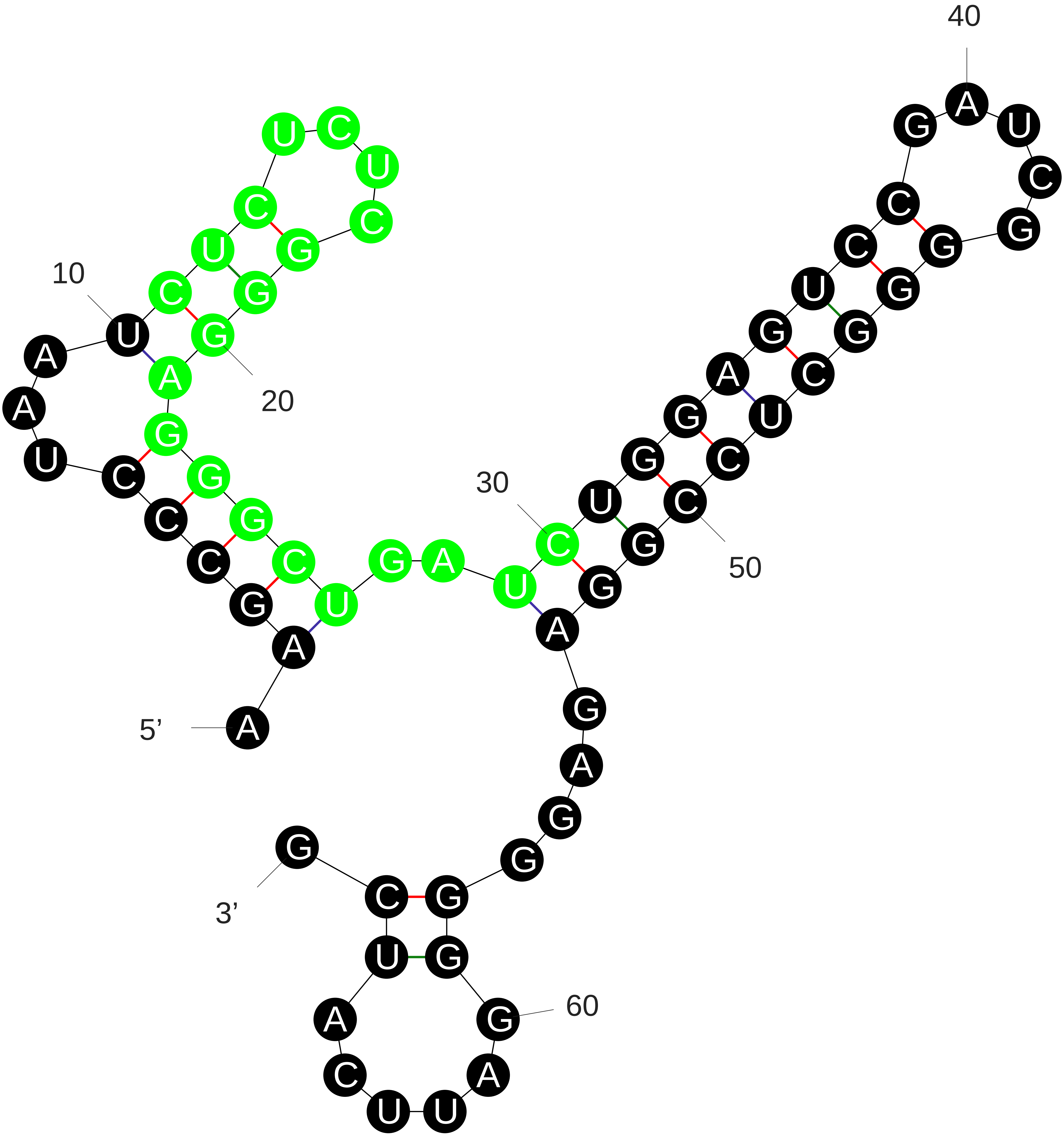

dG = -29.20 [Initially -29.20] Ta-miR068-5p

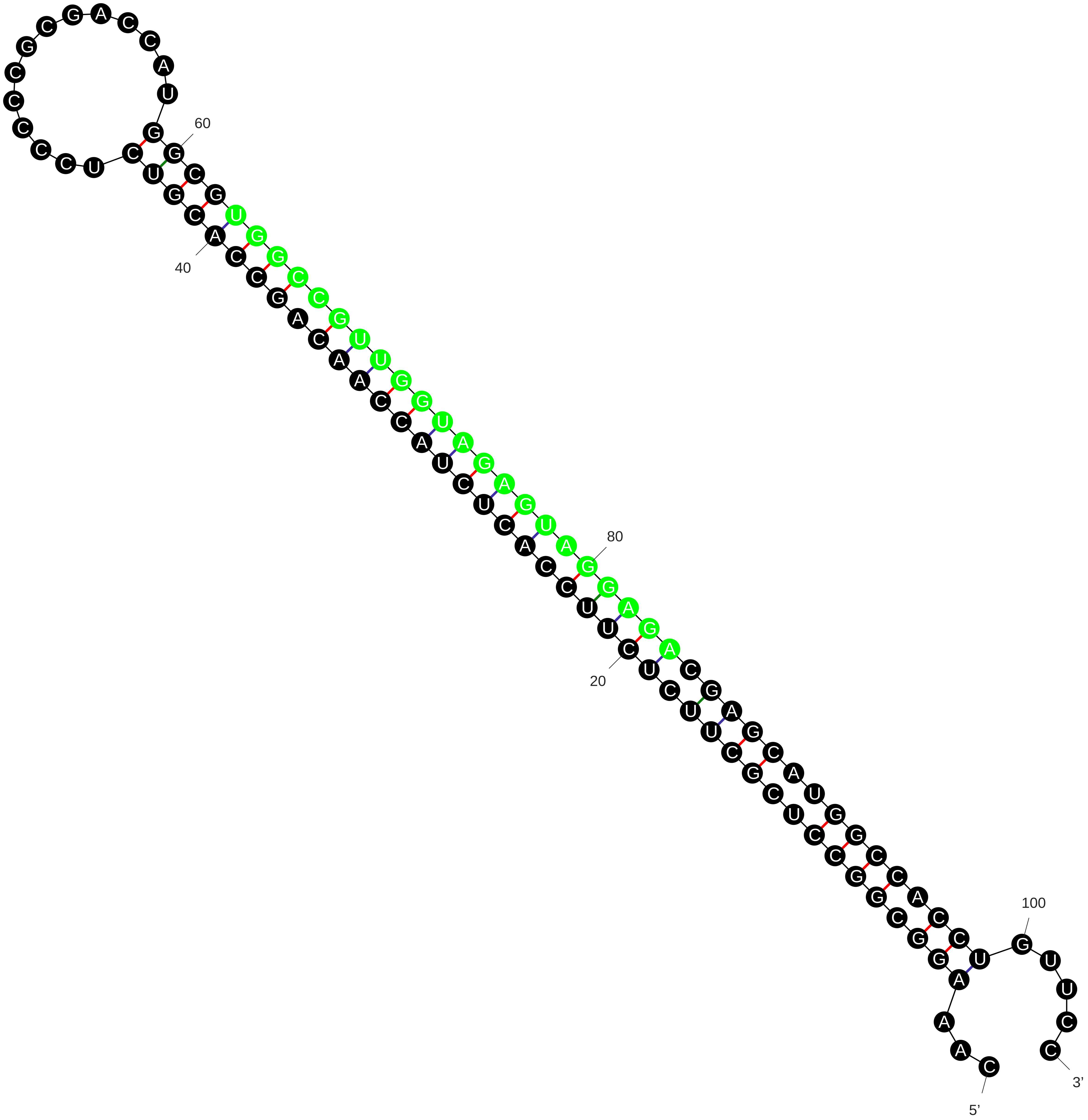

dG = -59.70 [Initially -59.70] Ta-miR057-1-3p

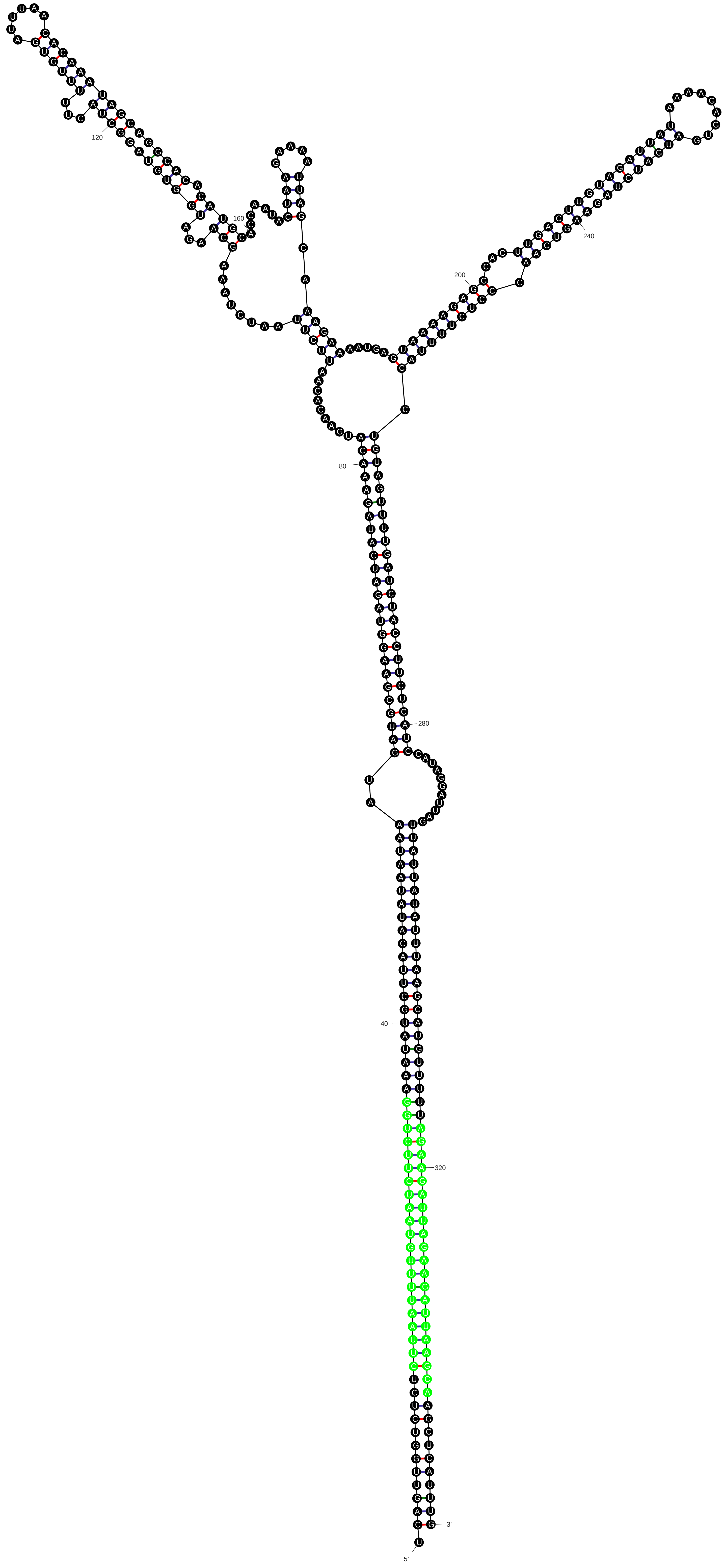

dG = -116.04 [Initially -122.90] Ta-miR007-5p/-3p

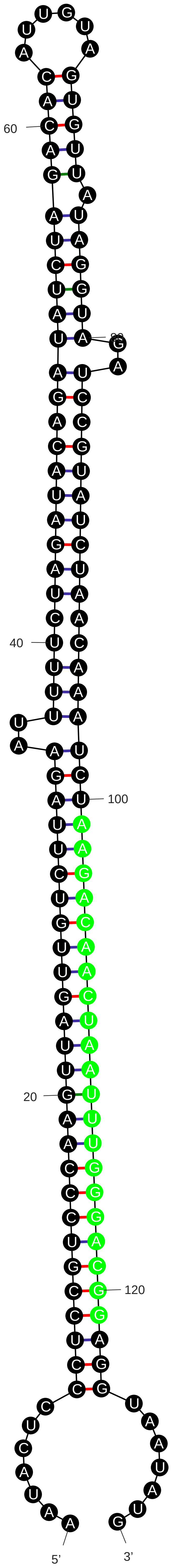

dG = -65.80 [Initially -65.80] Ta-miR158-3p

Output of sir_graph (©)
mfold_util 4.6

Created Mon Apr 22 10:17:43 2013

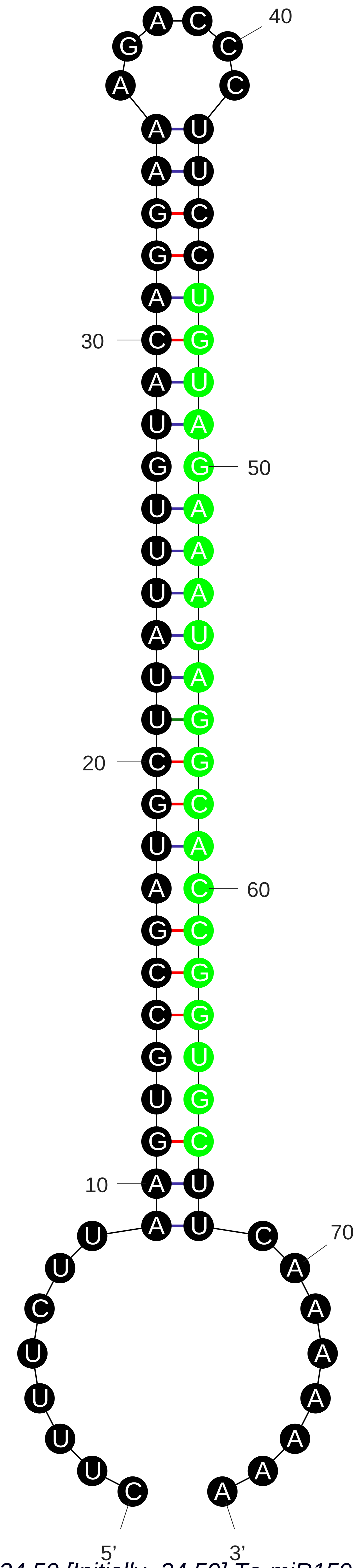

dG = -34.50 [Initially -34.50] Ta-miR159-3p

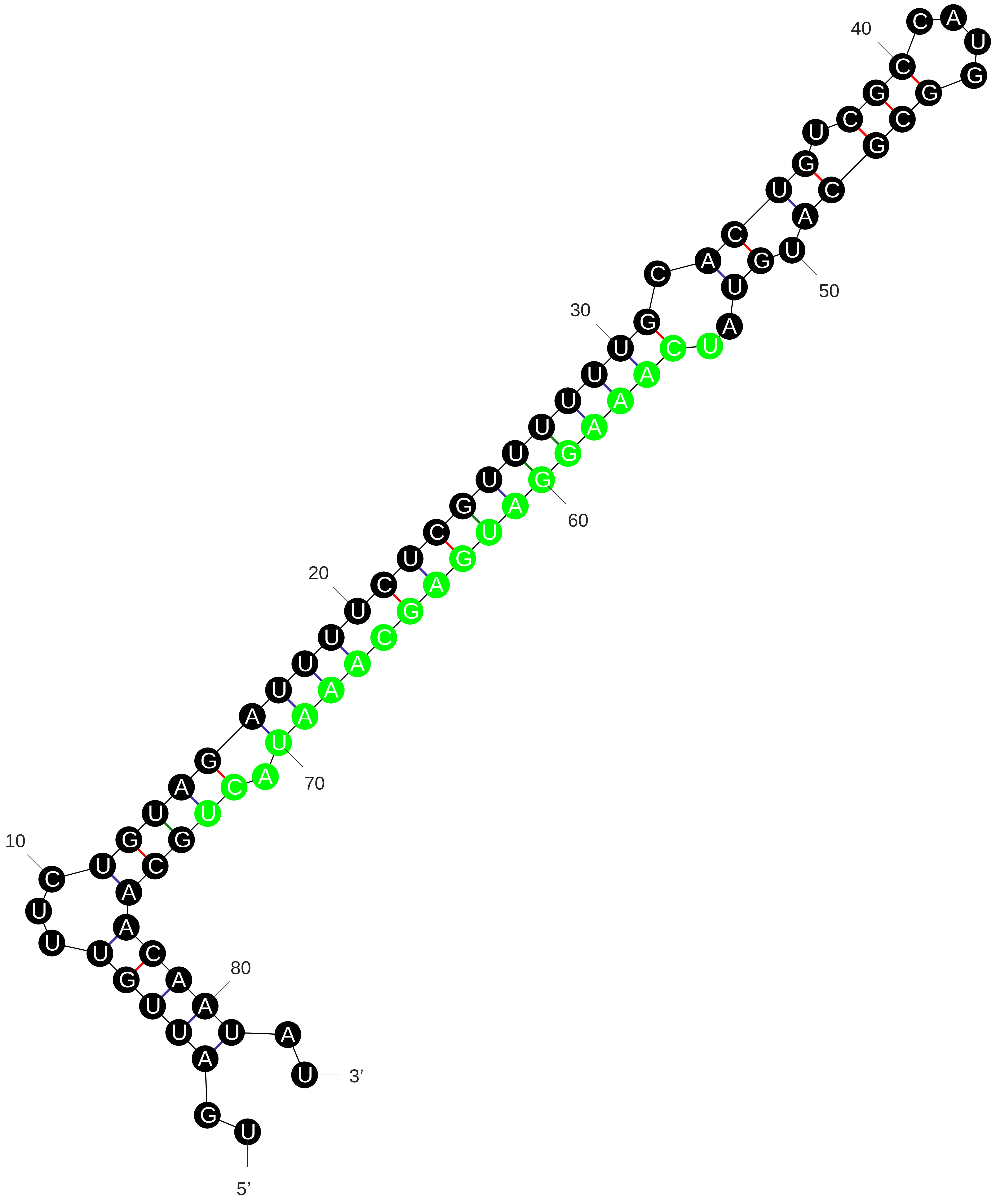

dG = -26.70 [Initially -26.70] Ta-miR033-3p

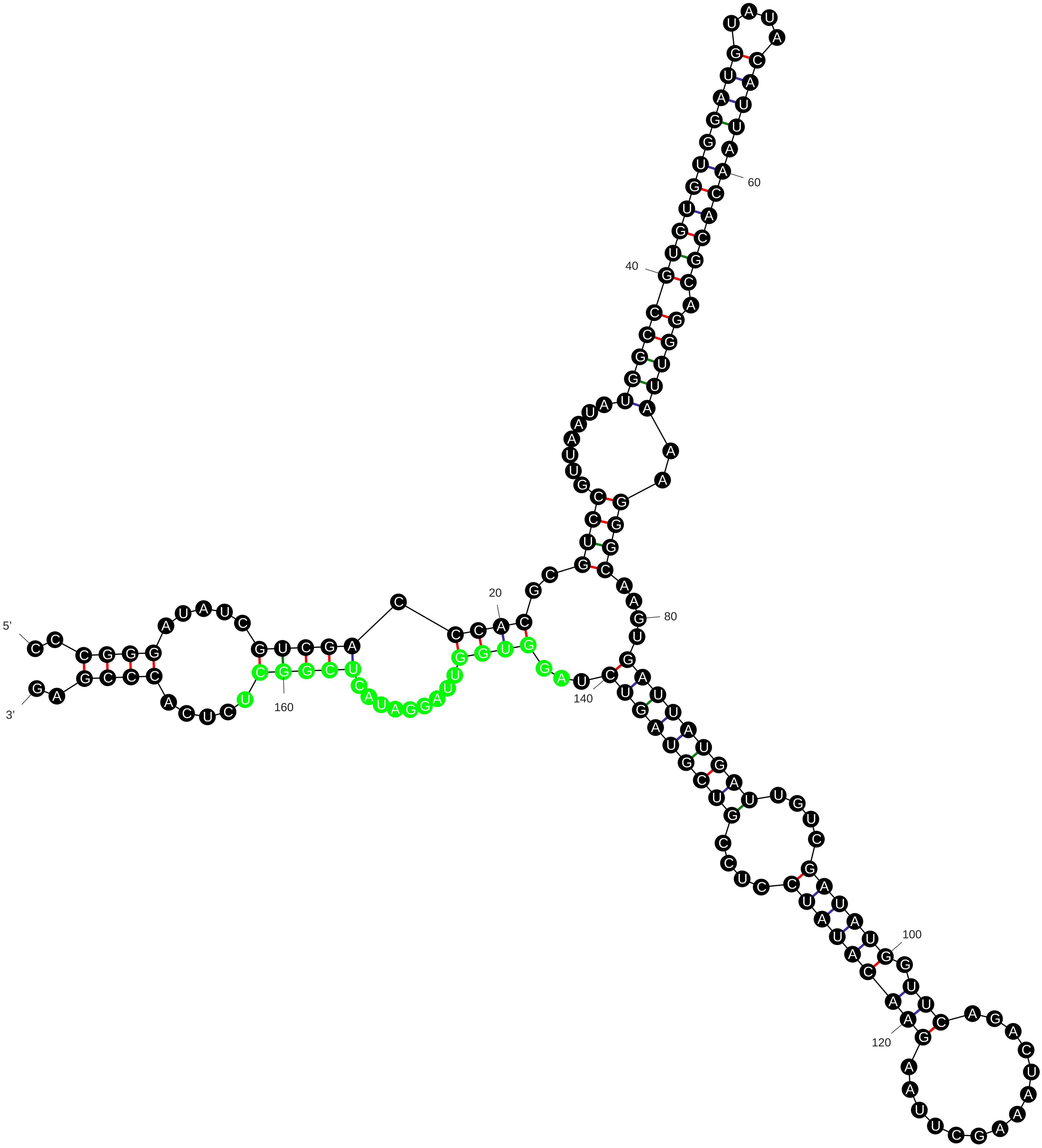

dG = -46.65 [Initially -50.20] Ta-miR053-3p

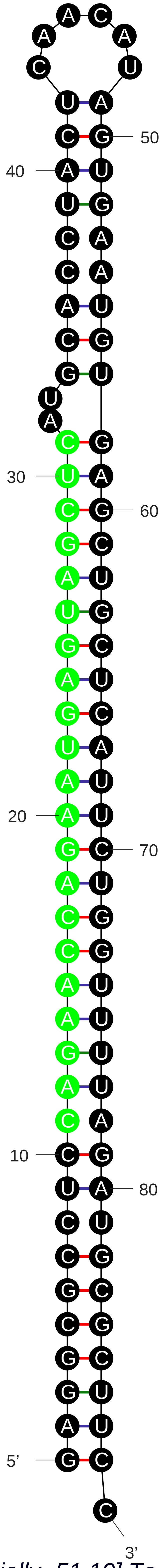

dG = -51.10 [Initially -51.10] Ta-miR039-5p
